# The gastrointestinal microbiome of browsing goats (*Capra hircus*)

**DOI:** 10.1371/journal.pone.0276262

**Published:** 2022-10-17

**Authors:** Vera Guerra, Igor Tiago, Aitana Aires, Catarina Coelho, João Nunes, Lígia O. Martins, António Veríssimo

**Affiliations:** 1 Department of Life Sciences, Center for Neuroscience and Cell Biology, University of Coimbra, Coimbra, Portugal; 2 Centre Bio R&D Unit, Association BLC3—Technology and Innovation Campus, Lagares da Beira, Oliveira do Hospital, Portugal; 3 Department of Life Sciences, Centre for Functional Ecology–Science for People and the Planet, University of Coimbra, Coimbra, Portugal; 4 FitoLab, Laboratory for Phytopathology, Instituto Pedro Nunes, Coimbra, Portugal; 5 Instituto de Tecnologia e Química Biológica António Xavier, Universidade Nova de Lisboa, Oeiras, Portugal; Universitat Autonoma de Barcelona, SPAIN

## Abstract

Despite the growing interest in the ruminants’ gastrointestinal tract (GIT) microbiomes’ ability to degrade plant materials by animal husbandry and industrial sectors, only a few studies addressed browsing ruminants. The present work describes the taxonomic and functional profile of the bacterial and archaeal communities from five different gastrointestinal sections (rumen, omasum-abomasum, jejunum, cecum and colon) of browsing *Capra hircus*, by metabarcoding using 16S rRNA genes hypervariable regions. The bacterial communities across the GITs are mainly composed of *Bacillota* and *Bacteroidota*. *Prevotella* was the leading bacterial group found in the stomachs, *Romboutsia* in the jejuna, and *Rikenellaceae_RC9_gut_group*, *Bacteroides*, *UCG-010_ge*, *UCG-005*, and *Alistipes* in large intestines. The archaeal communities in the stomachs and jejuna revealed to be mainly composed of *Methanobrevibacter*, while in the large intestines its dominance is shared with *Methanocorpusculum*. Across the GITs, the main metabolic functions were related to carbohydrate, amino acid, and energy metabolisms. Significant differences in the composition and potential biological functions of the bacterial communities were observed among stomachs, jejuna and large intestines. In contrast, significant differences were observed among stomachs and jejuna verse large intestines for archaeal communities. Overall different regions of the GIT are occupied by different microbial communities performing distinct biological functions. A high variety of glycoside hydrolases (GHs) indispensable for degrading plant cell wall materials were predicted to be present in all the GIT sections.

## Introduction

Rumen microbiome is primarily known for its ability to convert plant materials into volatile fatty acids, which their hosts then use as an energy source. Ruminants’ importance as a source of human food (milk and meat) leads to the rapid growth of animal husbandry and higher production of greenhouse gases. This has contributed to the growing knowledge about the rumen microbiome to e.g., improve feed conversion efficiency [1], and animals’ health [2], and mitigate methane emissions [3]. The ruminants microbiome’s ability to digest plants with high fibre content resulted in a large number of metagenomic studies, given the interest of the biobased industries in discovering microorganisms and lignocellulolytic enzymes to apply to their industrial processes [4–6].

The microbiome associated with small and large intestines is poorly known, and the majority of the studies have been performed in feedlot or grazing ruminants, rather than in wild or browsing ruminants. While grazers feed on undifferentiated low-growing vegetation that is poor in lignin, like grass, browsers tend to include in their diet leaves, and shoot from woody plants, like shrubs [7]. The consumption of plants of different lignocellulosic compositions was suggested to lead to different microbiomes’ composition and to the expression of different genes encoding carbohydrate-active enzymes (CAE) [8, 9].

A study performed on the faecal microbiome of wild browsers (i.e., giraffe, impala, and kudu) has revealed a higher potential to digest high fibre forages and reduce energy loss producing less enteric gas, like methane, than the microbiome of grazing goats [10]. Another study, this performed on rumen microbiome of domestic yaks feeding on alpine meadows with different amounts of shrub coverage, showed an increased abundance of bacteria with a higher ability to convert and harvest energy as the shrub coverage increases, indicating an improvement in the dietary energy utilization [11]. Results like these suggest that ruminants with a browsing diet are likely to have a more diverse microbiome able to digest a higher diversity of substrates and improve feed conversion efficiency.

In the present study the bacterial and archaeal populations present in the various areas of the gastrointestinal tract (GIT) of browsing goats (*Capra hircus*), raised in different shrubby areas of the Centre of Portugal, were analysed and compared taxonomically and functionally. This study aimed to obtain an insight into the GIT bacterial and archaeal diversity of Portuguese browsing *Capra hircus*, to understand how taxonomic and functional composition vary across the GIT, and analyse the diversity of putative genes encoding enzymes involved in the lignocellulose degradation of the shrubby vegetation.

## Materials and methods

### Collection of microbial gastrointestinal tract samples

Samples of gastrointestinal tract (GIT) contents were collected from five compartments, rumen (Ru), omasum and abomasum (OA), jejunum (Je), cecum (Ce) and colon (Co) from three adult female goats (*Capra hircus*). Samples were maintained at 4°C and processed in less than 24 h. The GITs were kindly donated after the goats been slaughtered in an abattoir at S. Paio de Gramaços, Chamusca da Beira, Central Portugal; with license and veterinarian control number B 06. The abattoir was working in accordance to the legal animal care and sanitary guidelines, namely Portuguese Law n° 113/2019, and European Regulation (CE) n° 1099/2009.

All goats grew up practicing browsing on bushlands and pine forests; goat G1 in Lajeosa village of Oliveira do Hospital County, goat G2 in Penhas Douradas and goat G3 in Loriga, both villages of Seia County, Portugal. For these browsing areas were constated a similar shrubby vegetation mainly composed of gorse (*Ulex europaeus*), broom (*Cytisus* sp.), “carqueja” (*Genista tridentata*) and some rockrose (Cistus *ladanifer*), species previously identified in these types of lands on the National Portuguese Forest Inventory [12] and chemically characterised [13].

### Metagenomic DNA extraction

The different samples of the GITs were kept on ice during DNA extraction and about 1g of homogenised sample of each GIT compartment was used. For the metagenomic DNA extraction was used the PowerMax^®^ Soil DNA Isolation Kit (MoBio^®^ Laboratories Inc., Carlsbad, CA, USA). The extraction process was carried out as explaned in the manufacturer’s procedures, except in the lyses step. In this step two cycles of 15 min each of -70°C and 70°C were performed. Each metagenomic DNA sample was analysed by 1% gel electrophoresis and purified using the same kit. The purified step also required adaptations: a solution of chloroform:isoamilic alcohol (24:1, v/v) (fisher) was added to the DNA samples and these were blended and incubated at 4°C for 20 min.

The purified DNA preparations were quantified by UV-Vis (Nanodrop 2000c spectrophotometer, Thermo Scientific) at 260 nm, concentrated in a centrifugal vacuum concentrator (SpeedVac SPD140 Vaccum Concentrator Kit, Thermo Scientific Thermo) and stored at -20°C.

### 16S rRNA gene sequencing

The fifteen samples of purified metagenomic DNA were sequenced in an Illumina Miseq V2 platform at the Genoinseq Laboratory, Cantanhede, Portugal (https://www.cnc.uc.pt/en/services).

To determine the bacterial and archaeal diversity the hypervariable regions V3-V4 and V4-V5 of the bacterial and archaeal 16S rRNA genes were amplified. The set of primers used in these amplifications for domain Bacteria were 357wF 5’-CCTACGGGNGGCWGCAG-3’ and 926wR 5’-CCGTCAATTYMTTTRAGTTT-3’ [14] and for domain Archaea were 517F 5’-GCYTAAAGSRNCCGTAGC-3’ and 909R 5’-TTTCAGYCTTGCGRCCGTAC-3’ [15].

### Bioinformatic and data analysis

Raw sequence data quality-control, clustering, and taxonomic analysis were processed with mothur v.1.44.1 software package (https://mothur.org/) [16]. Sequence reads with low quality, ambiguous bases, and chimeras were excluded. The obtained high-quality sequences were clustered into operational taxonomic units (OTUs) on a 97% sequence similarity level and taxonomically assigned using Silva reference files SSU ARB-SILVA database v.138 [17, 18]. Coverage, richness index (Chao) and α-diversity indices (Inverse Simpson, Shannon) were also calculated through the mothur software package. For better visualization, relative abundance histograms of the phyla and family classification were drawn, using the Microsoft^®^ Excel^®^ 2016 v.2202, and a heatmap of the relative abundance of the dominant bacterial genera was created using the STAMP v.2.1.3 software [19].

Beta-diversity was determined by the principal coordinate analysis (PCoA), based on the Bray-Curtis index, using MicrobiomeAnalyst (https://www.microbiomeanalyst.ca/) [20]. To determine the average dissimilarity and the relative abundance of the major contributors to the differences observed in the bacterial and archaeal communities, a similarity percentages (SIMPER) analysis was performed using PAST v.4.0 [21].

All the raw sequence data were deposited at the NCBI database with the accession number PRJNA806670 (https://www.ncbi.nlm.nih.gov/sra/PRJNA806670).

### Functional gene prediction

PICRUSt2 (Phylogenetic Investigation of Communities by Reconstruction of Unobserved States, v.2.0) [22] was used to predict the functional gene content of each bacterial and archaeal community of the fifteen GIT samples. The same clustered 16S rRNA gene sequence reads, previously used for taxonomic classification, were used for PICRUSt2 analysis. The predicted functions were pre-calculated in KEGG (Encyclopedia of Genes and Genomes) database and summarised into KEGG pathways at Levels 2, 3, and 4. The glycoside hydrolases (GHs) were identified in CAZy database (http://www.cazy.org, accessed on 26 May 2021) for further analysis.

The differences in predicted functions between GIT compartments were observed through principal component analysis (PCA) using the Canoco v.4.5 software package [23] and through box plots using STAMP v.2.1.3 software [19].

### Statistical analysis

The normal distribution was tested using the Shapiro-Wilk and Kolmogorow-Smirnov tests in R (v.x64 4.1.1). Once the data did not display a normal distribution, the comparison of the fifteen samples was carried out using non-parametric tests.

Kruskal-Wallis H-test and Tukey-Kramer post-hoc tests were chosen to compare and assess significant differences (at p<0.05) in the relative abundance of taxa and KEGG pathways, among GIT samples.

## Results

### Richness and diversity analysis of the bacterial communities

The high-quality reads retained for bacterial populations were 694 844, after quality filtering, chimera removal and the disregard of OTUs with ≤ 10 sequences. OTUs were grouped based on ≥ 97% sequence identity. Across the goats’ GIT, 3 721 bacterial OTUs were identified.

In general, higher numbers of OTUs and values of richness (Chao index) were found in the stomachs (Ru and OA) and large intestines (Ce and Co) (Table 1). The highest values of bacterial alpha-diversity (Inverse Simpson index and Shannon index) were confirmed in the stomachs and large intestines and the lowest values of alfa-diversity were found in jejuna (Je).

**Table 1:** 16S rRNA gene sequences, richness and alpha-diversity estimates of *Bacteria* at the GIT of three goats.

Samples	Reads	OTUs	Invsimpson	Shannon	Chao	Coverage (%)
G1Ru	98 007	593	74.3	5.1	669.6	99.9
G2Ru	44 732	777	145.3	5.6	846.5	99.8
G3Ru	29 222	1 400	72.9	5.8	1 566.8	99.2
G1OA	43 263	1 143	37.8	5.1	1 321.8	99.5
G2OA	29 946	1 230	156.6	5.9	1 386.2	99.3
G3OA	38 195	1 450	92.5	5.9	1 605.3	99.5
G1Je	41 333	644	13.3	4.1	711.7	99.8
G2Je	35 361	622	7.3	3.3	741.4	99.6
G3Je	29 059	758	50.7	4.8	901.1	99.4
G1Ce	75 235	1 153	106.6	5.5	1 309.6	99.8
G2Ce	29 226	1 086	88.5	5.5	1 227.1	99.4
G3Ce	41 469	1 180	109.4	5.7	1 387.3	99.6
G1Co	70 540	228	59.1	4.5	247.3	100.0
G2Co	54 141	1 080	97.4	5.6	1 181.1	99.8
G3Co	35 115	1 104	107.5	5.6	1 210.6	99.6

G: goat; Ru: rumen; OA: omasum-abomasum; Je: jejunum; Ce: cecum; Co: colon; Invsimpson: inverse Simpson index

All the coverage estimates were ≥ 99.2% (Table 1), inferring that most of the bacterial diversity was detected.

### Phylogenetic analysis of the bacterial communities

**Fig 1:**
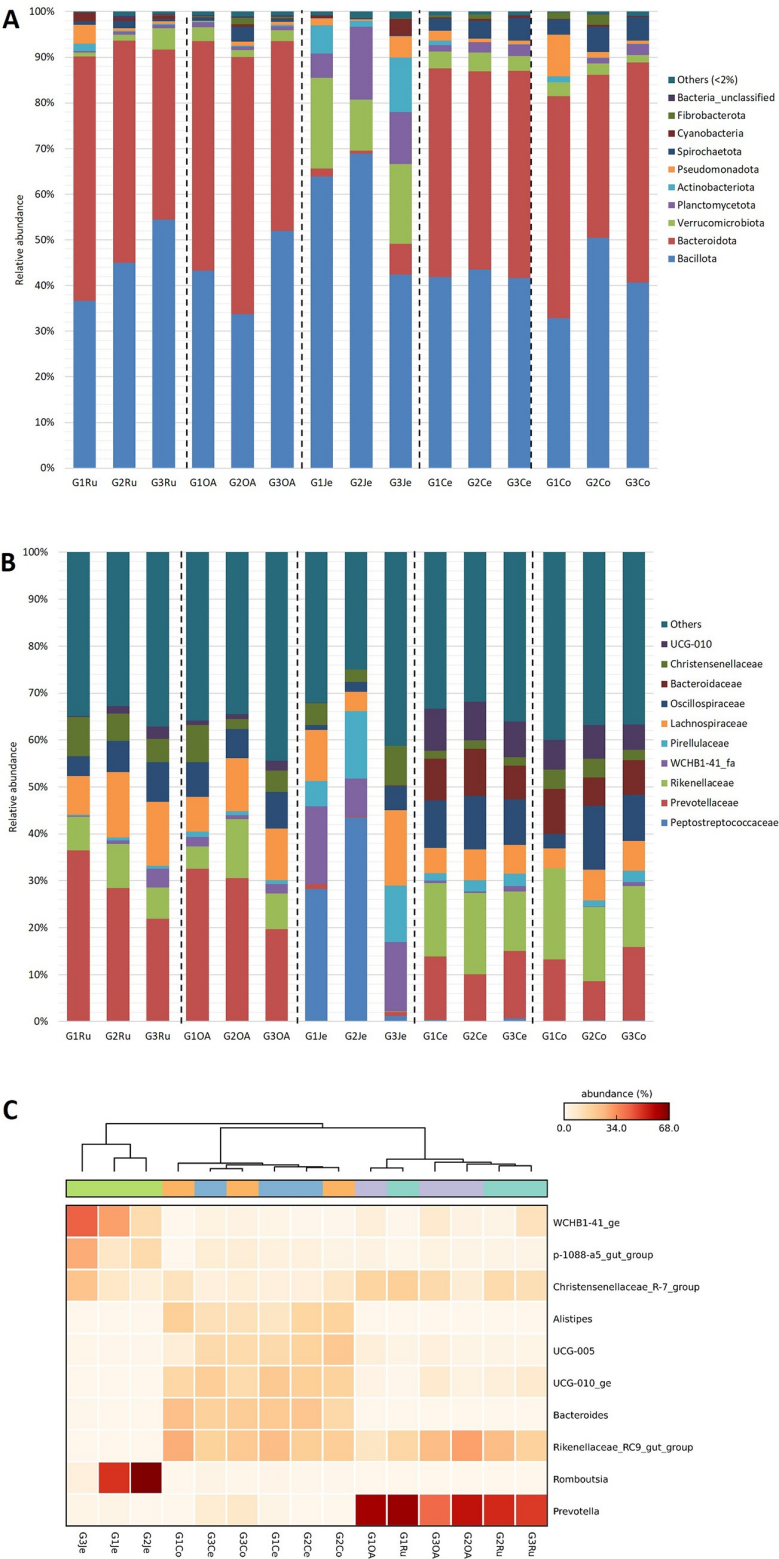
Distribution of the ten most abundant bacterial phyla (A), families (B) and genera (C) in *Capra hircus* GITs. Ru: rumen; OA: omasum + abomasum; Je: jejunum; Ce: cecum; Co: colon; G: goat.

Across the goats’ GITs were identified 3 721 bacterial OTUs assigned to 18 phyla, 37 classes, 88 orders, 150 families, and 309 genera. Fig 1A shows the phyla distribution in each one of the GIT compartments of the three goats. The most prevalent phyla (average relative abundance ≥ 5% in at least one GIT compartment) were *Bacillota*, *Bacteroidota*, *Verrucomicrobiota* and *Planctomycetota*. *Bacillota* and *Bacteroidota* were the predominant phyla in the stomachs and large intestines, comprising 81.5–93.6% of the total number of sequences per sample and 78.6% (2 924) of the OTUs (Fig 1A). The relative abundances of *Bacillota* were high (32.9–68.9%) in all the GIT compartments achieving their highest values in the jejuna. *Bacteroidota* members were abundant in the stomachs and large intestines (35.7–56.3%), but not in the jejuna (0.6–6.7%) where members of this phylum were significantly (p<0.05) lower. The highest abundances of *Verrucomicrobiota* (11.3–19.9%), and *Planctomycetota* (5.3–15.9%) were observed in the jejuna. In all areas, *Bacillota* members represent the most abundant OTUs (1 966, 52.8%), followed by *Bacteroidota* (958, 25.8%), *Pseudomonodata* (230, 6.2%), *Verrucomicrobiota* (195, 5.2%), *Actinobacteriota* (97, 2.6%), *Spirochaetota* (87, 2.3%), *Planctomycetota*, and *Cyanobacteria* (both 34, 0.9%).

One hundred and fifty families were identified among the GIT compartments. The ten most abundant families are shown in Fig 1B. In the stomachs, the dominant family was *Prevotellaceae* (20.6–36.5%), followed by *Lachnospiraceae* (7.6–14.4%), and *Rikenellaceae* (4.9–12.3%), *Oscillospiraceae* (4.2–8.9%), and *Christensenellaceae* (2.1–8.4%). In the jejuna, the most abundant families were *Peptostreptococcaceae* (1.2–48.2%), *WCHB1-41_fa* (8.9–16.4%), *Pirellulaceae* (5.3–15.9%), *Lachnospiraceae* (4.6–15.3%), and *Christensenellaceae* (2.9–7.3%). In the large intestines, the most abundant were *Rikenellaceae* (12.1–17.9%), *Prevotellaceae* (7.9–15.5%), *Oscillospiraceae* (2.8–12.9%), *Bacteroidaceae* (5.7–9.4%), *UCG-010* (5.3–7.9%), *Lachnospiraceae* (3.9–6.2%), and *Christensenellaceae* (1.5–6.6%).

The relative abundances of *Prevotellaceae* (20.6–36.5%) were significantly higher (p<0.05) in the stomachs than in the jejuna and large intestines. The relative abundances of *Peptostreptococcaceae* (1.2–48.2%), *WCHB1-41_fa* (8.9–16.4%), and *Pirellulaceae* (5.3–15.9%) were significantly higher (p<0.05) in the jejuna than in the stomachs and large intestine. The abundances of *Oscillospiraceae* (2.8–12.8%), *Bacteroidaceae* (5.7–9.4%, p<0.05), and *UCG-010* (5.3–7.9%, p<0.05) were higher in the large intestine than in the stomachs and jejuna. Furthermore, *Rikenellaceae* (12.8–17.9%) showed significantly higher (p<0.05) relative abundances in the colon than in the stomachs and jejuna. The abundances of *Lachnospiraceae* (3.9–15.3%) and *Christensenellaceae* (1.5–8.4%) decreased across the GIT.

Three hundred and nine genera were identified among the GIT compartments. The ten most abundant genera are shown in Fig 1C. The four most dominant genera detected in the stomachs were *Prevotella* (12.9–30.7%), *Rikenellaceae_RC9_gut_group* (6.9–11.3%), and *Christensenellaceae_R-7_group* (2.0–8.3%). *Prevotella* was significantly higher (p<0.05) in the stomachs than in the jejuna and large intestines. *Rikenellaceae_RC9_gut_group* was significantly more abundant (p<0.05) in the stomachs than in jejuna. In the jejuna, the four abundant genera were *Romboutsia* (1.2–45.4%), *WCHB1-41_ge* (8.9–16.4%), *p-1088-a5_gut_group* (4.7–9.3%) and *Christensenellaceae_R-7_group* (2.9–7.3%). The first three were significantly more abundant (p<0.05) in the jejuna than in the other GIT compartments. In the large intestines, the dominant genera were *Rikenellaceae_RC9_gut_group* (7.5–10.5%), *Bacteroides* (5.7–9.4%), *UCG-010_ge* (5.3–7.9%), *UCG-005* (1.8–8.2%) and *Alistipes* (3.7–6.9%). *Rikenellaceae_RC9_gut_group* (6.7–10.5%), as observed in the stomachs, showed higher (p<0.05) relative abundances in the large intestine than in jejuna. The abundances of *Bacteroides* (5.7–9.4%), *UCG-010_ge* (5.3–7.9%) and *Alistipes* (3.7–6.9%) were significantly higher (p<0.05) in the large intestines, than in the stomachs and jejuna. Also, *UCG-005* (5.3–8.2%) showed significantly higher (p<0.05) abundances in the large intestines than in the stomachs and jejuna but was not significantly higher in the colons than in omasum-abomasums. *Christensenellaceae_R-7_group* (2.0–8.3%) occurred in all the compartments with a decreasing abundance across the GIT.

### Beta-diversity analysis of the bacterial communities

**Fig 2:**
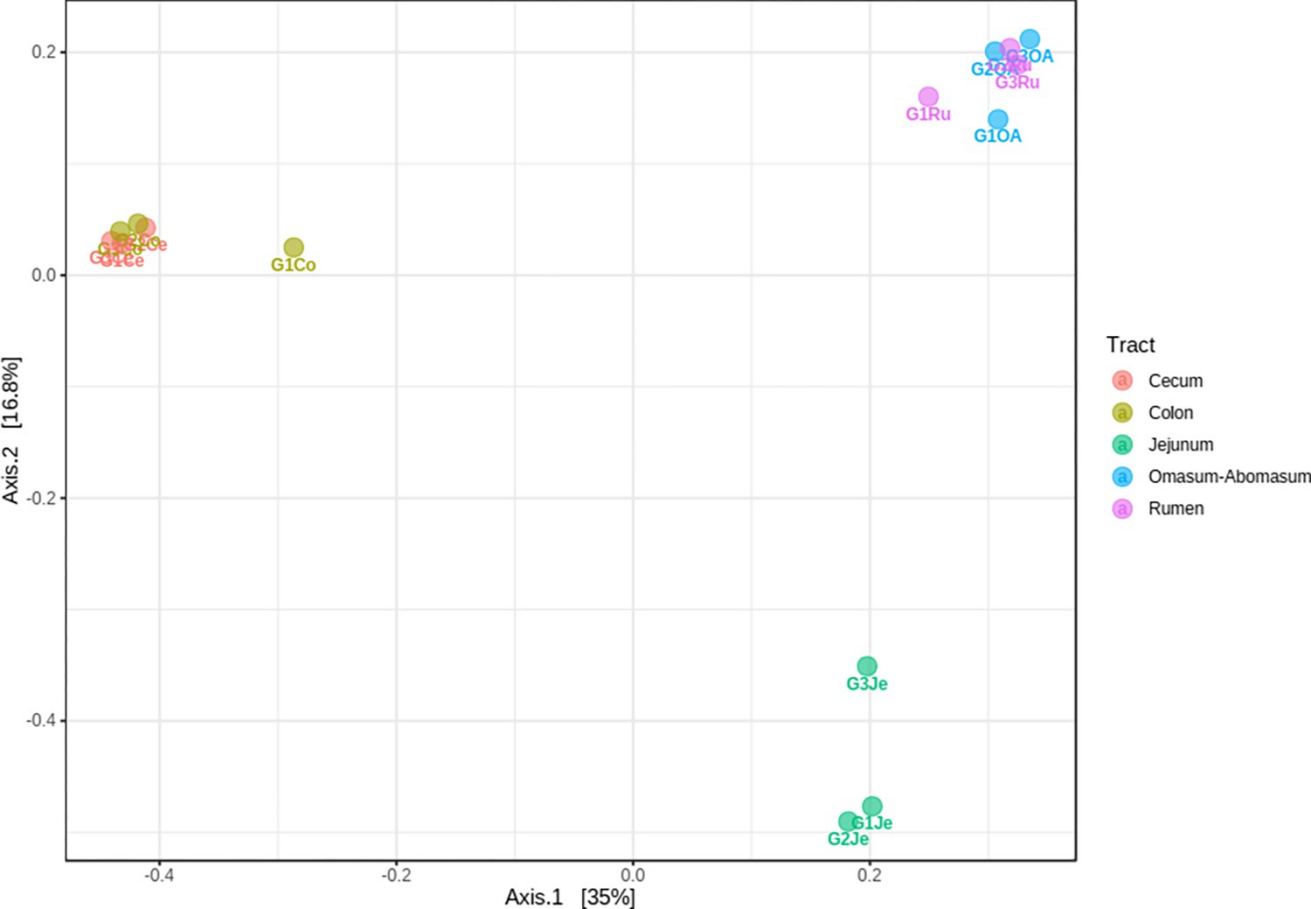
Comparison of the bacterial diversity present in the *Capra hircus* GITs. The differences among samples were based on the relative abundance of OTUs. Ru: rumen; OA: omasum + abomasum; Je: jejunum; Ce: cecum; Co: colon; G: goat.

The comparative analysis of all samples’ diversity (beta-diversity) is shown in Fig 2. The analysis showed a clear difference among the bacterial communities present in the stomachs, jejuna, and large intestines, as shown by Axis 1 (35.5% of variation) and Axis 2 (17.9% of variation). However, the bacterial communities from the adjacent GIT sections, Ru-OA and Ce-Co, grouped indicate the presence of similar populations.

Similarity percentages (SIMPER) analysis was also performed to compare the bacterial communities of the GITs (S2 Table) and to identify the major contributors in observed variations (S1 Table). The analysis showed the highest values of overall average dissimilarity between stomachs/jejuna (Ru: 78.3%, OA:78.8%), jejuna/large intestines (Ce: 86.0%, Co: 85.4%), and stomachs/large intestines (65.4–68.2%). Comparing the stomachs/jejuna communities, the decrease in abundance of members of the genus *Prevotella* and the increased abundance of *Romboutsia* members were the major contributors to the differences observed (29–30.2% of cumulative contribution). While for the differences in jejuna/large intestines communities were the decreased abundance of *Romboutsia* and *WCHB1-41_ge* (21.4–21.8%). Comparing the stomachs/large intestines communities, the decreased abundance of *Prevotella* and increased abundance of *Bacteroides*, contributed to 20.9–22.4% of the dissimilarities.

### Functional analysis of the bacterial communities

The functional profile of the bacterial communities present in each section of the goat’s GITs was predicted using PICRUSt2, based on 16S rRNA data and the Kyoto Encyclopedia of Genes and Genomes (KEGG) database, was used to predict the functional profile of the bacterial communities present in each section of the goats GITs. A total of 6 706 KEGG orthologs (KOs) were predicted to be present in all the bacterial communities, with more than half of the predicted genes participating in metabolic functions (53.8–57.4%, Level 1 KEGG, S2 Fig).

**Fig 3:**
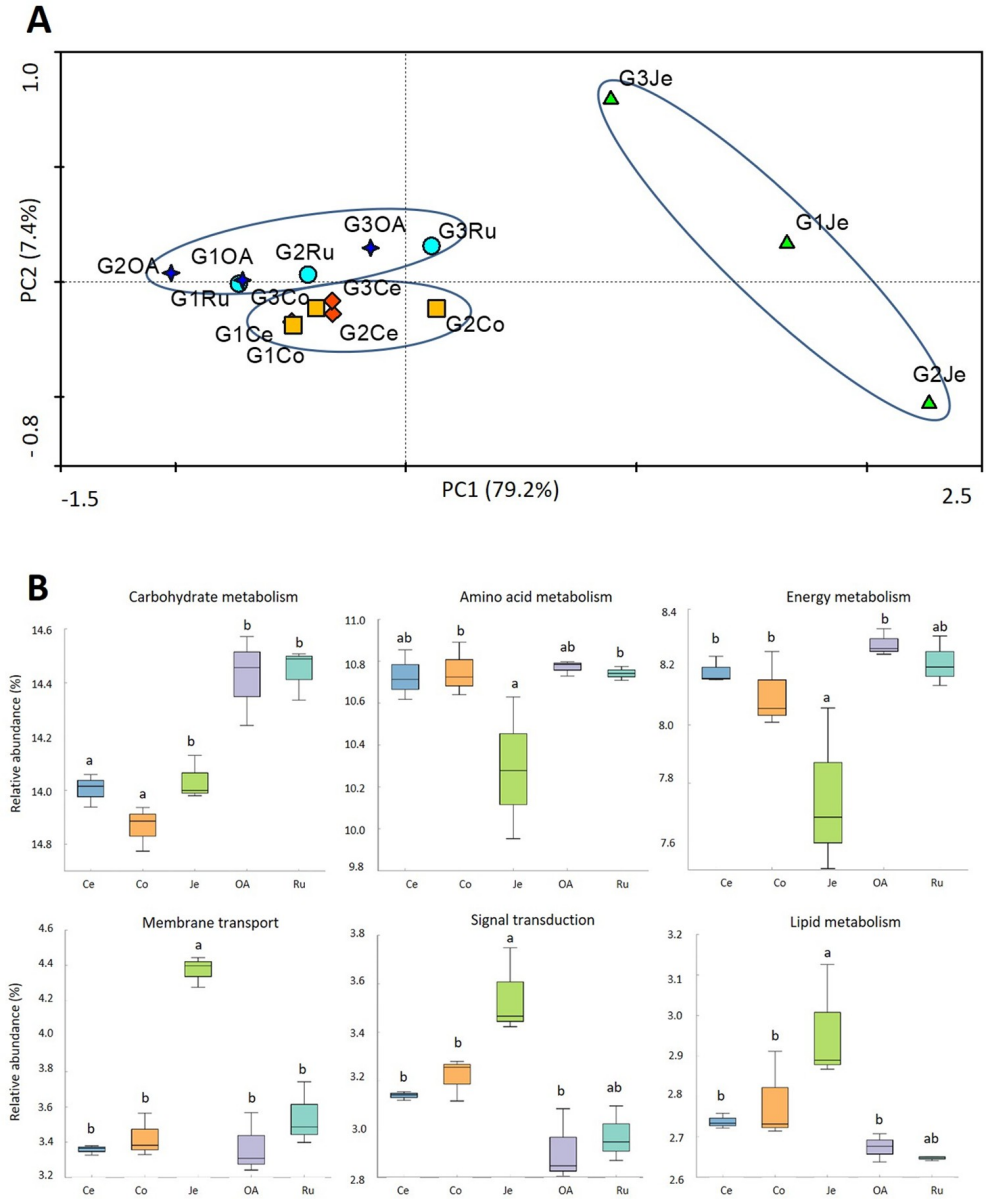
(A) Comparison of the bacterial functional diversity at Level 2 KEGG present in the *Capra hircus* GITs. (B) Comparison of the six most abundant bacterial gene families among the rumen (Ru), omasum-abomasum (OA), jejune (Je), cecum (Ce) and colon (Co) bacterial communities. Different superscripts were significantly different (P < 0.05).

At the Level 2 KEGG, from the 50 gene families predicted, 47 gene families were predicted to be present in all the bacterial communities comprising functions such as carbohydrates metabolism (13.8–14.6%), amino acid metabolism (10–10.9%), and energy metabolism (7.5–8.3%) (S3 Fig). A principal component analysis (PCA) based on the relative abundance values of the Level 2 KEGG pathways (Fig 3A) revealed that bacterial communities of the stomachs (Ru and OA) tend to share biological functions, such as the communities found in the large intestines (Ce and Co), while the predicted functions for bacterial communities in the jejuna (Je) were clearly distinguished (PC1: 79.2%).

In the stomachs, were observed the highest abundance of genes associated with carbohydrate metabolism with a significant decrease throughout the GIT (p < 0.05) (Fig 3B). The abundance of gene families related to amino acid and energy metabolisms decreased significantly (p < 0.05) in jejuna, compared with the high values verified in the other areas. On the opposite, the abundances of the gene families involved in the membrane transport, signal transduction, and lipid metabolism were significantly higher (p < 0.05) in jejuna than in the remaining GIT compartments. Only between jejuna and the colons, for signal transduction and lipid metabolism, was not verified a significant difference (p > 0.05).

### Carbohydrate-active enzymes in the bacterial communities

**Fig 4:**
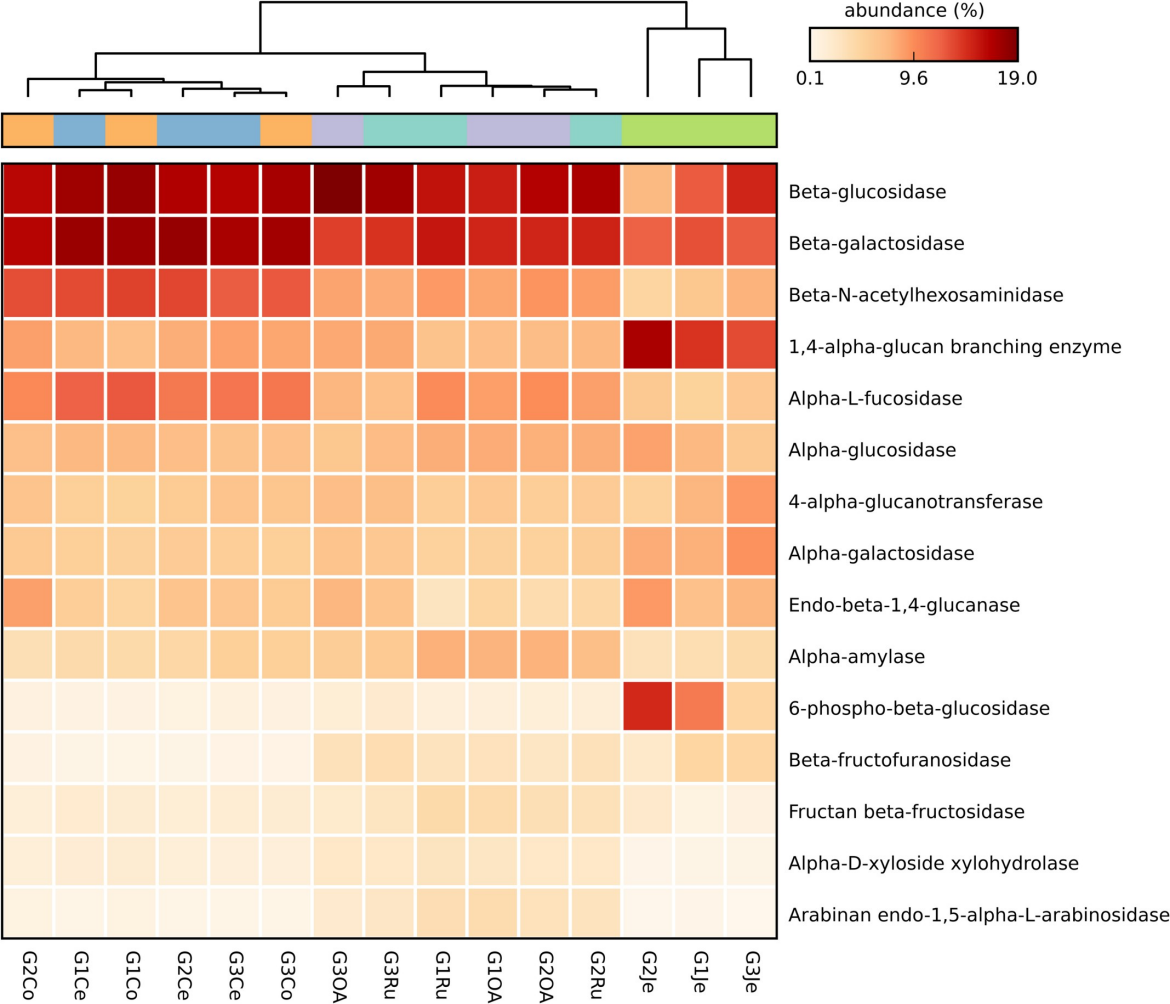
Heatmap of the 15 most abundant bacterial GHs predicted genes participating in carbon metabolism across the *Capra hircus* GITs. The heatmap was constructed with the relative abundances of the functional Level 4 KEGG pathways. Ru, rumen; OA, omasum + abomasum; Je, jejunum; Ce, cecum; Co, colon.

Eighty-six glycoside hydrolases (GHs) were predicted to be expressed by the bacterial communities of the goats’ GIT (S3 Table). The 15 most abundant GHs are shown in Fig 4, and comprise cellulases, hemicellulases, and oligosaccharide-degrading enzymes. The most abundant GHs in stomachs and large intestines of the goats were β-glucosidase (EC:3.2.1.21, 12.1–15.7%) and β-galactosidase (EC:3.2.1.23, 11.2–14.8%), while in the jejuna, 1,4-alpha-glucan branching enzyme (EC:2.4.1.18, 10.8–13.1%) and 6-phospho-β-glucosidase (EC:3.2.1.86, 3.7–11.3%) were very abundant. High abundance of β-N-acetylhexosaminidase (EC:3.2.1.52, 610.4–11.1%) and α-L-fucosidase (EC:3.2.1.51, 8.1–10.2%) in large intestine samples was also observed.

To predict the different contributions of the GHs across GIT regions, a principal component analysis (PCA) was performed (S6 Fig). Thus, while α-amylase (EC:3.2.1.1) was more representative in the Ru and OA, in the Ce and Co was β-N-acetylhexosaminidase. In Je, 6-phospho-beta-glucosidase was the main contributor to the differentiation between the tract regions.

Among the total predicted GHs, 15 are known to be indispensable to metabolizing cellulose and hemicellulose (S3 Table).

### Richness and diversity analysis of the archaeal communities

After all the low-quality reads, chimeras, and OTUs with ≤ 10 sequences have been disregarded, 340 804 reads were retained, representing the archaeal populations present across the goats’ GIT. Based on ≥ 97% sequence identity between reads, 73 OTUs were identified.

The numbers of OTUs and richness values in the rumens, jejuna, and large intestines samples, were higher than those observed in the omasum-abomasum samples (Table 2). The values of the Inverse Simpson and Shannon indices showed higher values in the large intestines than in the stomachs and jejuna samples, except in G3Ce and G3Co (Table 2).

**Table 2:** 16S rRNA gene sequences, richness and alpha-diversity estimates of *Archaea* at the GIT of three goats.

Samples	Reads	OTUs	Invsimpson	Shannon	Chao	Coverage (%)
G1Ru	51 109	28	1.2	0.4	30.0	100.0
G2Ru	15 342	28	1.3	0.5	39.0	99.9
G3Ru	18 256	21	1.1	0.2	33.0	100.0
G1OA	5 321	14	1.4	0.5	14.2	100.0
G2OA	5 948	16	1.5	0.7	21.0	99.9
G3OA	12 395	17	1.1	0.3	19.0	100.0
G1Je	25 991	21	1.2	0.4	31.0	100.0
G2Je	32 376	29	2.3	1.1	30.5	100.0
G3Je	37 398	23	1.1	0.2	33.0	100.0
G1Ce	25 126	24	2.2	0.9	52.0	100.0
G2Ce	20 781	24	2.2	0.9	25.2	100.0
G3Ce	18 902	24	1.0	0.1	31.0	100.0
G1Co	37 133	46	2.4	1.1	46.0	100.0
G2Co	25 539	21	2.3	1.0	21.8	100.0
G3Co	9 187	17	1.0	0.1	38.0	99.9

G: goat; Ru: rumen; OA: omasum-abomasum; Je: jejunum; Ce: cecum; Co: colon; Invsimpson: inverse Simpson index.

The coverage values were ≥ 99.9% for all GIT sections, indicating that the sequences were representative of the archaeal diversity (Table 2).

### Phylogenetic analysis of the archaeal communities

Seventy-three archaeal OTUs were assigned to 2 phyla, 3 classes, 3 orders, 3 families, and 6 genera. Fifty-eight OTUs were assigned to the *Euryarchaeota*, while 15 OTUs were assigned to the *Halobacterota*. Only members of the phylum *Euryarchaeota* were detected in the stomachs and jejuna (S1A Fig). In the large intestines, both *Euryarchaeota* (Ce: 45.6–56.1%; Co:44.2–55.8%) and *Halobacterota* (Ce: 43.9–54.9%; Co:44.2–55.8%) representatives were found. The exception was in the goat G3, where *Euryarchaeota* was the only phylum identified.

At the family level, the dominant families were *Methanobacteriaceae* and *Methanocorpusculaceae*. In the stomachs and jejuna were only identified members of *Methanobacteriaceae*. In the large intestines, members of *Methanobacteriaceae* (Ce: 45.6–56.1%; Co:44.2–55.8%) and *Methanocorpusculaceae* (Ce: 43.3–54.4%; Co:44.2–54.6%) shared high representativeness, except in goat G3 where only members of *Methanobacteriaceae* were detected (S1B Fig).

**Fig 5:**
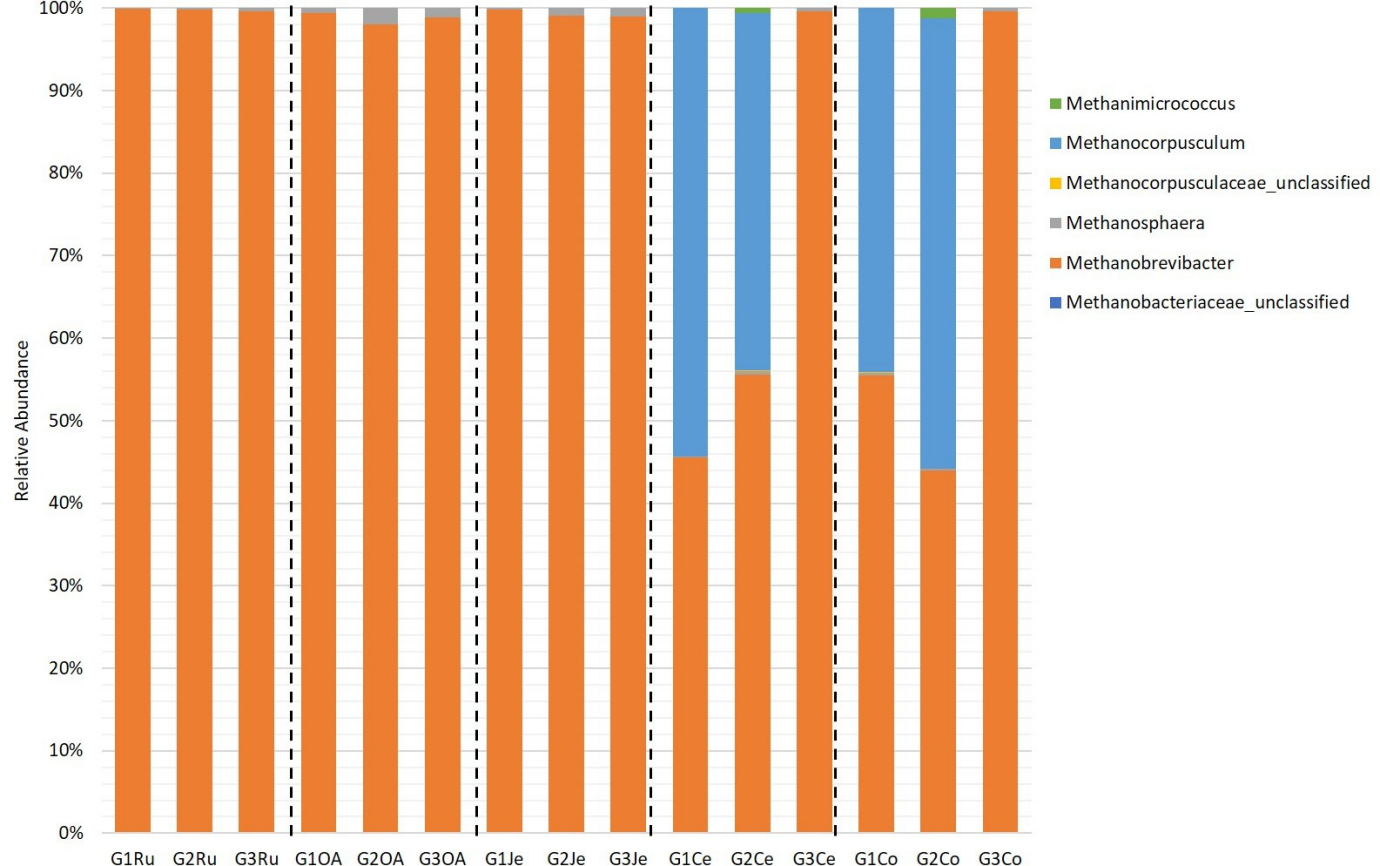
Distribution of the total archaeal genera in *Capra hircus* GITs. Ru: rumen; OA: omasum + abomasum; Je: jejunum; Ce: cecum; Co: colon; G: goat.

Six archaeal genera were identified: *Methanobrevibacter*, *Methanocorpusculum*, *Methanosphaera*, *Methanimicrococcus*, unclassified *Methanocorpusculaceae*, and *Methanobacteriaceae* (Fig 5). In the stomachs and jejuna, were detected *Methanobrevibacter* (98.0–99.9%) and *Methanosphaera* (0.1–1.0%). In the large intestines, the predominant genera were *Methanobrevibacter* (44.0–55.6%) and *Methanocorpusculum* (44.1–54.6%), except in the large intestine of the goat G3, where 99.6% of the sequences were assigned to the genus *Methanobrevibacter*.

### Beta-diversity analysis of the archaeal communities

**Fig 6:**
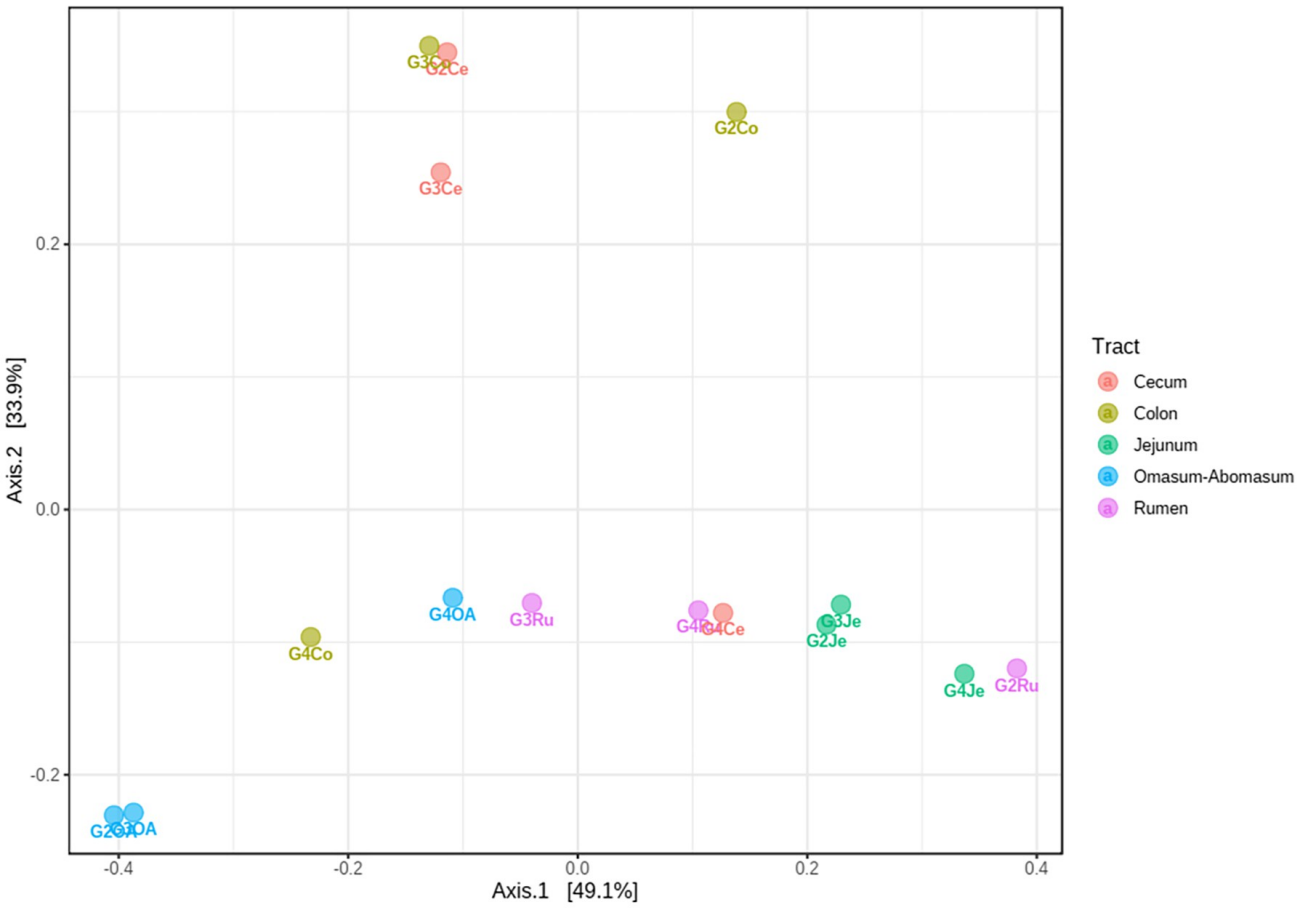
Comparison of the archaeal diversity present in the *Capra hircus* GITs. The differences between samples were based on the relative abundance of OTUs. Ru: rumen; OA: omasum + abomasum; Je: jejunum; Ce: cecum; Co: colon; G: goat.

The archaeal diversity of all samples was compared through a PCoA based on the Bray-Curtis index at the OTUs level (Fig 6). This showed that the Ce and Co archaeal communities differed from the other GIT sections (accounted for 34% of the variation, Axis 2) except G3Ce and G3Co. Also, differences between the OA and the Ru and Je archaeal communities were observed (with 49% of variation, Axis 1).

SIMPER analysis showed the highest values of overall average dissimilarity between large intestines/stomachs (32.9–33.6%), and large intestines/jejuna (32.9–33.5%) (S5 Table). The differences between large intestines/stomachs and between large intestines/jejuna may be explained by the decreased abundance of the genus *Methanobrevibacter* and the increased abundance of *Methanocorpusculum* (98–99.4% of the cumulative contribution, S6 Table), except in G3 where the relative abundance of *Methanobrevibacter* remained higher (Fig 5).

### Functional analysis of the archaeal communities

The analysis to predict the functional profile of the archaeal communities revealed a total of 1 706 KEGG orthologs. More than half of the predicted genes participated in metabolic functions (56.3–58.1%, Level 1 KEGG, S4 Fig). There were identified 43 gene families (at Level 2 KEGG). The most abundant were related to carbohydrate metabolism (14.7–16.9%), amino acid metabolism (10.2–10.7%), and membrane transport (5.4–8.5%) (S5 Fig).

**Fig 7:**
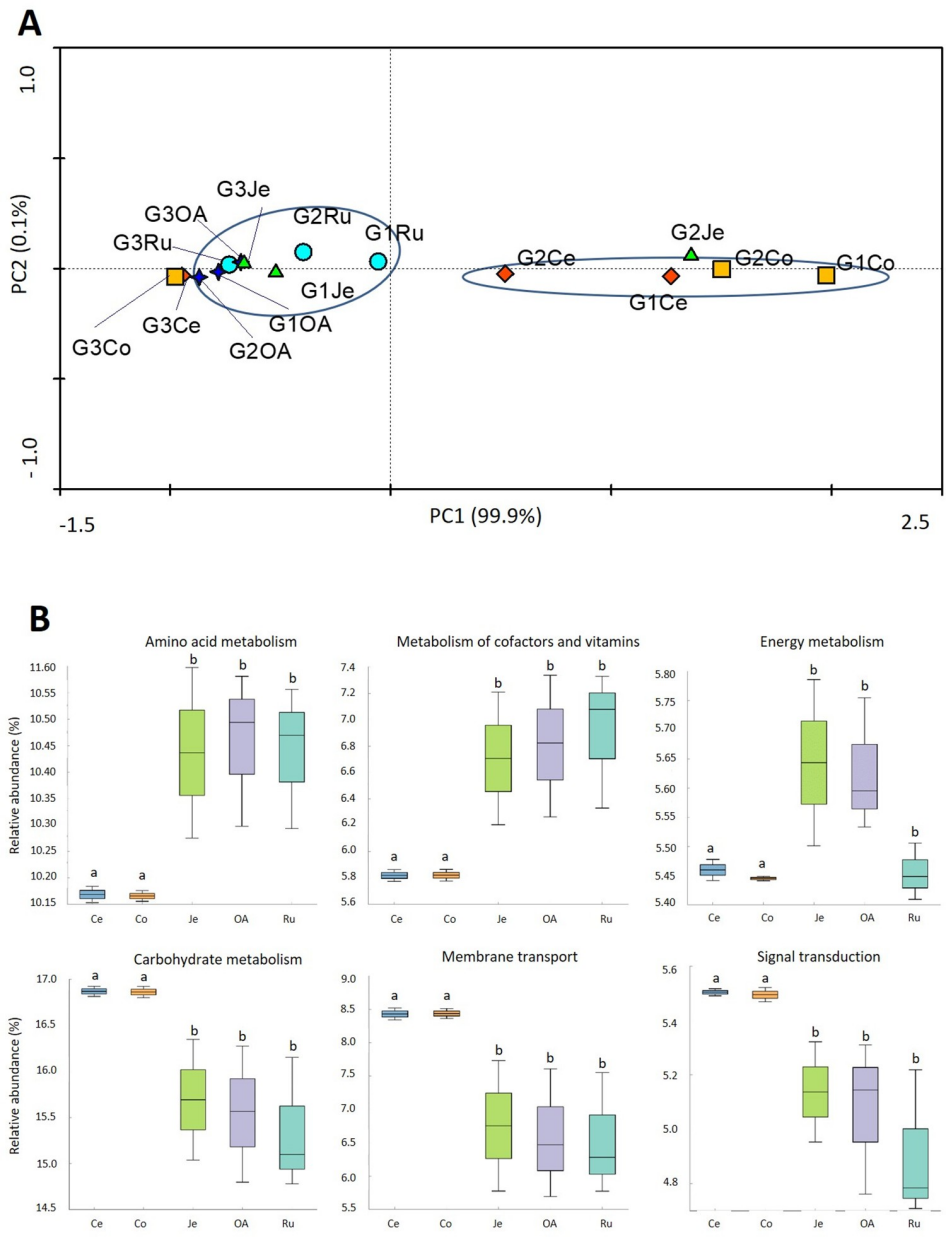
(A) Samples distribution based on the archaeal functional diversity at Level 2 KEGG present in the *Capra hircus* GITs. (B) Comparison of the six most abundant archaeal gene families with significant differences (p < 0.05) among rumen (Ru), omasum-abomasum (OA), jejune (Je), cecum (Ce) and colon (Co) archaeal communities. Different superscripts were significantly different (P < 0.05).

The PCA on the relative abundances of Level 2 KEGG gene families (Fig 7A) revealed that the samples of the stomachs (Ru and OA) and jejuna (Je) tend to join (PC2: 0.1%), whereas the samples of the large intestine (Ce and Co) were clearly distinguished from the others accounting with a variation of 99.9% (PC1). Only the samples G3Ce, G3Co, and G2Je, were found in the opposite clusters. If these outlier samples were excluded from our analysis, the archaeal communities present in the stomachs and jejuna had a higher relative abundance (p < 0.05) of genes related to amino acid metabolism, metabolism of cofactors and vitamins, and energy metabolism than in the large intestines (Fig 7B). In the large intestines, a higher relative abundance (p < 0.05) of genes associated with carbohydrate metabolism, membrane transport, and signal transduction were observed, than in the upper gut samples.

### Carbohydrate-active enzymes in the archaeal communities

Nine CAE, mainly glycosyltransferases (GT), were predicted for the archaeal communities present in goats’ GIT (S4 Table). Endo-beta-1,4-glucanase, and α-amylase were the only identified GHs. Endo-beta-1,4-glucanase was identified in all the GIT compartments (2.5–33.3%) while α-amylase was only present in large intestines (10.1–11.9% and 10–11.7%, respectively). However, this GH was not predicted in the large intestine of goat G3.

## Discussion

### Diversity, richness, and taxonomic composition of bacterial microbiome across the goat gastrointestinal tract

This study investigated the bacterial and archaeal communities’ composition and potential biological functions across the GITs of browse-feed *Capra hircus* from the Centre Inner Region of Portugal. The free-ranging animals were reared by local goatherds feeding on shrubby vegetation mainly composed of gorse (*Ulex europaeus*), broom (*Cytisus* sp.), “carqueja” (*Genista tridentata*) and some rockrose (Cistus *ladanifer*) [12] species chemically characterised as rich in cellulose and xylan [13].

Analysis of the bacterial diversity showed that the bacterial community in the jejuna (Je) was significantly less diverse than in the stomachs (Ru and OA) and large intestines (Ce and Co). Similar results were obtained in other goats [24], sheep [25], and dairy cattle [26], where it was noted that the characteristics of each GIT region had a strong influence in the structure and composition of the bacterial community. Different physical and chemical conditions—morphological structure, pH, gut motility, oxygen concentration, redox potential, availability of nutrients, and host secretions—found in each GIT region [27] related to their functional differences [28] can explain the observed differences in bacterial diversity. The lower richness and diversity observed in jejuna could result from the lower pH due to host secretions, such as bile acid, or to the irregular reception of digesta and its short permanence in the small intestine [29]. Moreover, the increase of bacterial richness and diversity in the large intestine could be the result of favorable environmental conditions for the bacteria that escaped from the stomachs and small intestine, to grow and digest the remaining digestible content [30, 31]. Indeed, *Rikenellaceae_RC9_gut_group* and *Christensenellaceae_R-7_group* very abundant in stomachs were also found in the large intestine.

The bacterial microbiome differed significantly between stomachs, jejuna and large intestine, however, in adjacent compartments, like rumen/omasum-abomasum and cecum/colon, the bacterial communities tend to be similar as revealed by PCoA and Kruskal-Wallis analysis. Analogous results were obtained in previous studies using other goats [24, 32], sheep [25], and dairy cattle [26]. Analysis of the bacterial composition of stomachs and large intestine microbiome, at the phylum level, showed a high abundance of *Bacillota* and *Bacteroidota*, whereas, in the jejuna, *Bacillota*, *Verrucomicrobiota* and *Planctomycetota* were the most abundant phyla. *Bacillota* members are known for their role in fibre degradation, mainly in cellulose degradation, into VFA [33] then used by the host to fulfill their energy needs. *Bacteroidota* participates primarily in the degradation of complex soluble polysaccharides and proteins [34, 35] that, along with host enzymes, improve host accessibility to nutrients, and allow their storage. *Bacillota* members are mainly acetate and propionate producers, while *Bacteroidota* members are butyrate producers [36]. *Bacillota* and *Bacteroidota* have frequently been pointed out as dominant phyla in ruminants GIT. Variations in the *Bacillota*:*Bacteroidota* ratio, are possibly due to differences in diet, species, seasonal, environment, or methods of analysis [26, 37–39]. Previous studies on the microbiome composition of the small intestine in ruminants reported *Pseudomonodata* as the second most abundant phylum [25, 26, 37, 40], not being expected such high relative abundances of *Verrucomicrobiota* and *Planctomycetota* in the analysed goats’ jejuna. *Verrucomicrobiota* was reported to participate in polysaccharide degradation, like cellobiose [28, 41], as well as, in the methane oxidation to methanol [42]. *Planctomycetota* was identified as involved in the degradation of biopolymers, like chitin [43].

The most abundant genera in the stomachs were *Prevotella*, *Rikenellaceae_RC9_gut_group* and *Christensenellaceae_R-7_group*, which reached up to 44.5% in some of these GIT compartments. In previous studies on goats [32, 44], members of *Prevotella* and families *Rikenellaceae* and *Christensenellaceae* were also found predominant in the rumen. In the present study, *Prevotella* alone reached up to 30.3% of the total abundance. Indeed, members of this genus were typically observed in higher numbers in stomachs (26.7–49.9%) than in other regions of the GIT of ruminants [25, 37, 39]. Different members of the genus *Prevotella* are known for their capability to degrade non-cellulolytic polysaccharides (hemicelluloses, mainly xylans, pectin, starch, lignans) and proteins, although some cellulolytic enzymes may also be produced [45–47]. *Prevotella* dominance in rumen bacterial communities is thought to be related to their genetic and functional diversity [48, 49], the reason why they may be indispensable players in the transformation of the ingested feed by ruminants.

Despite the high abundances of *Rikenellaceae_RC9_gut_group* reported in the stomachs and large intestines of ruminants, its role is still unclear. Studies on the rumen microbiome of yaks [50] and cows [51] relate *Rikenellaceae_RC9_gut_group* to the degradation of structural carbohydrates; which may explain its significant presence in the stomachs of the goats analysed.

The *Christensenellaceae_R-7_group* was identified in the stomachs of yaks [10, 52], sheep and goats [53] browsing on shrub-coverage pasture. Members of the *Christensenellaceae_R-7_group* are capable of degrading carbohydrates, amino acids, and carboxylic acids into acetate and butyrate [54].

Members of the family *Lachnospiraceae* represent the second most abundant bacterial family in the stomachs. Members of this family were reported at higher concentrations in sheep and cows with rich-fibre diets [9, 55]. They are known for their capability to degrade xylan, cellulose, and starch and produce acetate and butyrate [55, 56]. As butyrate producers, members of *Lachnospiraceae* and *Christensenellaceae* have been associated with the intestinal health preventing intestinal inflammation [54, 57] and stimulating energy expenditure and fatty acid oxidation by their hosts [58] contributing to weight management.

In the large intestines, was observed a co-dominance of five genera; *Rikenellaceae_RC9_gut_group*, *Bacteroides*, *UCG-010_ge*, *UCG-005*, and *Alistipes*, that together reached an abundance of 39.4%. These groups were also reported in faecal samples of bovines [59], musk deer [60] and alpine ungulate [61]. *Rikenellaceae_RC9_gut_group* and *Alistipes* were reported to be closely related bacteria belonging to the *Rikenellaceae* family [62]. *Alistipes* members can produce acetate and succinate and are frequently asaccharolytic [63], which may explain the emergence of this genera in the large intestine samples in the present study. *Bacteroides* members were known to help their hosts digest polysaccharides due to their capability to degrade them to monosaccharides [60], and to produce acetate, propionate, and succinate [64]. *UCG-010_ge*, and *UCG-005* belong to the order *Oscillospirales*, previously undissociated from the family *Ruminococcaceae*. Studies supported the participation of *UCG-010* in fibre degradation, mainly in cellulose, and in the biohydrogenation of dietary polyunsaturated fatty acids (PUFA) to saturated fatty acids (SFA) [65, 66]. Previous studies suggested the enhancement of fibre degradation by *UCG-005* members in ruminants feeding on high shrub coverage [10].

In stomachs, members of the *Prevotellaceae* family were mainly composed of members of genus *Prevotella*, while in large intestines this family was mainly composed by members of *Prevotellaceae_UCG_004*, *Prevotellaceae_UCG_003*, and *Prevotellaceae_UCG_001*. Previous studies also detected these groups in the rumen and intestines of ruminants feeding on high fibre content feed [65, 67, 68], thus, suggesting the existence of functional redundancy and contributing to further downstream feed fermentation in the intestines.

In the jejuna, the dominant groups of bacteria belonged to *Romboutsia*, *WCHB1-41_ge*, and *p-1088-a5_gut_group*. Alone, *Romboutsia* reached up to 45.4% of the total abundance. The dominance of *Romboutsia* in jejuna ruminants was also observed previously [38, 69]. Their metabolic capabilities linked to carbohydrate use, fermentation of amino acids and production of VFA was reported [70, 71]. This may indicate that jejunum bacteria also play an important role in the digestion of feed.

### Predicted functions and carbohydrate active enzymes of bacterial microbiome across the goat gastrointestinal tract

In the present study, the most abundant functional categories predicted are related to the metabolism of carbohydrates, amino acids, and energy; which are metabolic functions essential for bacterial growth, and consistent with other studies performed on goats [24], cows [26] and camels [30]. Significant differences in predicted bacterial functions between GIT regions provide evidence that the functional profile of the bacterial communities changes from one GIT region to another. The bacterial communities in stomachs seem to have a significant role in carbohydrate degradation, agreeing with a dairy cattle study [26]. From the present work, we can infer that genes related to amino acid metabolism and energy metabolism were present in high abundance in the bacterial communities of all GIT, except in the jejuna. We can hypothesize that the bacterial cells are degraded by the acid secretions in the abomasum, leading to a consequent lower abundance and diversity of bacteria with participation in carbohydrate, amino acid, and energy metabolism, in jejuna. Moreover, the bacterial communities in the jejuna appear to be more specialized in lipid digestion, vitamin production, and facilitating the absorption of nutrients by the host, as can be inferred by the higher number of genes related to membrane transport, signal transduction, and lipid metabolism. Contrary to other GIT compartments, the bacterial communities in large intestines seem to have a major role in protein and energy metabolism. However, this is not in agreement with other studies performed in other goats [24, 32] and also in camels [30] where lower abundances of genes related to amino acid metabolism were reported. We can hypothesize that bacterial communities in the large intestines may participate in the additional metabolization of proteins (i.e., microbial crude protein) and of polysaccharides probably escaped from upper gut digestion, suggesting the production of energy derived from the use of these substrates.

When analysing the diversity and abundance of the glycoside hydrolases (GHs) across the GIT goats, even though these showed to be similar in adjacent GIT sections (Ru/OA and Ce/Co), differences were observed between stomachs, jejunum, and large intestine. In the stomachs and large intestines, β-glucosidase (EC:3.2.1.21) and β-galactosidase (EC:3.2.1.23) genes were the most abundant. These enzymes have been reported as essential to the complete degradation of cellulose and xyloglucan, respectively [72, 73]. The β-N-acetylhexosaminidase (EC:3.2.1.52) and α-L-fucosidase (EC:3.2.1.51) genes, also at high abundances in large intestines, were reported to participate in chitin degradation [74] and xyloglucan degradation [72], respectively. Such as hypothetically proposed in other studies, the degradation of structural carbohydrates appears to occur in the large intestine of ruminants [2, 75, 76]. In jejuna, 1,4-alpha-glucan branching enzyme (EC:2.4.1.18) and 6-phospho-β-glucosidase (EC:3.2.1.86) genes were the most abundant. 1,4-alpha-glucan branching enzymes are known to be involved in starch and glycogen metabolism catalysing modifications in the structures of these polysaccharides [77], whereas 6-phospho-β-glucosidase has been reported to participate in cellulose degradation [78]. Also, the abundance of β-glucosidase and β-galactosidase genes in jejuna bacteria suggests their capability to metabolize these complex polysaccharides. Previous studies on ruminants’ small intestines microbiome identified GHs with cellulose and xylan breakdown [79, 80].

### Diversity, richness, and taxonomic composition of archaeal microbiome across the goat gastrointestinal tract

Analysis of archaeal diversity and richness across the GIT of the goats showed higher diversity in the large intestines suggesting a shift in the archaeal communities’ composition from small to large intestines. The loss of dominance of *Methanobrevibacter* in the stomachs and jejuna to a shared dominance of this genus with *Methanocorpusculum* in large intestines corroborates this observation. Previous studies performed on rumen and faecal samples of bovines [59, 81], sheep [82], and camels [83] also noted this effect. Still, a clear cause for this shift could not be identified. Furthermore, when analysing the archaeal community of the goat G3, a less diversity was observed and mentioned shift did not occur, as it was confirmed by the constant presence and similar relative abundance of the genera *Methanobrevibacter* (98.9–99.6%) and *Methanosphaera* (0.4–1.1%) in all GIT sections. A similar situation was also verified in dairy cows [84]. Concerning the presence of methanogens in GIT environments, *Methanocorpusculum* sp. dominance has mainly been reported in faeces of the hindgut of horses [85] and rhinoceros [86]. Common to all goats was the dominance of *Methanobrevibacter* in the archaeal community of stomachs and jejuna. A study performed on a global scale, by Henderson *et al*. [87], also verified the dominance of this genus when comparing the rumen and camelid foregut archaeal community of animals from different geographic regions with different diets.

### Predicted functions and carbohydrate active enzymes of archaeal microbiome across the goat gastrointestinal tract

The predicted functional diversity was in agreement with the observed archaeal diversity, suggesting the attribution of certain specific roles to certain groups or genera. In the same way that the dominant genera switched from small to large intestines, the dominant predicted functions changed accordingly. For example, major abundance of genes associated to the metabolism of amino acids, cofactors, vitamins, and energy in the stomachs and small intestine could be assigned to *Methanobrevibacter*, whereas in large intestines a major role in the metabolism of carbohydrates, membrane transport, and signal transduction can be attributed to *Methanocorpusculum* (Figs 5 and 7).

The composition and functions of archaeal communities varied across the GIT. Overall, the expected functions of archaeal communities were similar from animal to animal, despite the variation in the large intestines verified in one animal (goat G3, Fig 5). Although in herbivorous GITs, *Methanocorpusculum* has been only reported in faecal or large intestine samples [87–89], species from this genus were reported to use the same substrates (i.e., H_2_ and formate) that *Methanobrevibacter* species, for methanogenesis [90, 91]. A hypothesis proposed by others to explain the abundance of *Methanocorpusculum* [88] and *Methanobrevibacter* species [84] focuses on the availability of substrates for methanogenesis supplied by the hydrogen/formate producers with whom they may develop a symbiotic relationship.

The analysis of the predicted CAE genes, with the identification of two GHs, endo-β-1,4-glucanases, and alpha-amylases, suggests that members of the archaeal communities may participate in cellulose and starch degradation. However, the predicted diversity of GHs for bacteria was much higher than for archaea, suggesting the importance of bacterial populations in the degradation of these compounds.

## Conclusions

The present work allowed a comprehensive examination of the GIT microbiome of browsing goat *Capra hircus*. The differences found in the taxonomical abundance of the main bacterial groups found within each gastrointestinal tract section are a mere reflection of the significant differences observed in the structure of the bacterial communities among the GIT regions. The functional analysis predicted that these bacterial groups have a major role in carbohydrate degradation in the stomachs, in the lipid digestion, vitamin production, and absorption of nutrients in the jejuna, and in the protein and energy metabolisms in the large intestines. The prediction of GHs genes encoding lignocellulases in jejuna, ceca and colon, leads us to believe that these regions may deserve special attention as sources of bacterial enzymes with interest for biobased industries.

The taxonomical analysis of the archaeal communities revealed relevant differences between the jejuna and the large intestines. Archaeal communities in stomachs and jejuna seem to have a major role in the metabolism of amino acids, energy, cofactors and vitamins, while in the large intestines seem to have functions related to the metabolism of carbohydrates, membrane transport, and signal transduction. The few predicted GHs genes suggests a weak participation of these communities in the hydrolysis reactions.

The present study has revealed the heterogeneity in the taxa composition and functional capacities of the bacterial and archaeal communities across the GIT of browsing *Capra hircus*, and has provided information about the potential of these microbiomes as a source of lignocellulosic enzymes.

## Supporting information

S1 Fig Distribution of the total archaeal phyla (A) and archaeal families (B) in *Capra hircus* GITs. Ru: rumen; OA: omasum + abomasum; Je: jejunum; Ce: cecum; Co: colon; G: goat.
(TIF)

S2 Fig Distribution of the bacterial functions in the *Capra hircus* GITs predicted at Level 1 KEGG. Ru: rumen; OA: omasum + abomasum; Je: jejunum; Ce: cecum; Co: colon; G: goat.
(TIF)

S3 Fig Distribution of the bacterial functions in the *Capra hircus* GITs predicted at Level 2 KEGG. Ru: rumen; OA: omasum + abomasum; Je: jejunum; Ce: cecum; Co: colon; G: goat.
(TIF)

S4 Fig Distribution of the archaeal functions in the *Capra hircus* GITs predicted at Level 1 KEGG. Ru: rumen; OA: omasum + abomasum; Je: jejunum; Ce: cecum; Co: colon; G: goat.
(TIF)

S5 Fig Distribution of the archaeal functions in the *Capra hircus* GITs predicted at Level 2 KEGG. Ru: rumen; OA: omasum + abomasum; Je: jejunum; Ce: cecum; Co: colon; G: goat.
(TIF)

S6 Fig Comparison of the 15 most abundant bacterial GHs genes (Level 4 KEGG) in the *Capra hircus* GITs. Ru: rumen; OA: omasum + abomasum; Je: jejunum; Ce: cecum; Co: colon; G: goat.
(TIF)

S1 Table Overall average dissimilarity (in percentage) between the bacterial communities present in the *Capra hircus* GITs. (PDF)

S2 Table Similarity percentages (SIMPER) analysis between the stomachs/jejuna and large intestine/jejuna the bacterial communities in the *Capra hircus* GITs. Avg: average dissimilarity; Contrib. (%): percent contributed; Cumul. (%): cumulative percentage; Ru: rumen; OA: omasum + abomasum; Je: jejunum; Ce: cecum; Co: colon. Bold indicates the relative percentage of the bacterial genera that contributed most to the observed dissimilarity between samples.
(PDF)

S3 Table Sequence based classification of glycoside hydrolases (GHs) predicted through 16S gene sequences by PICRUSt2 in the bacterial communities of *Capra hircus* GITs. Not Classified (N.C.). *Enzymes reported among the main to be involved in the direct degradation of lignocellulose [73, 92].
(PDF)

S4 Table Sequence based classification of carbohydrate active enzymes (CAE) predicted through 16S gene sequences by PICRUSt2 in the archaeal communities of *Capra hircus* GITs. Glycoside Hydrolase (GH), Glycosyl transferase (GT), and Carbohydrate Binding Module (CBM).
(PDF)

S5 Table Overall average dissimilarity (in percentage) between the archaeal communities present in the *Capra hircus* GITs. (PDF)

S6 Table Similarity percentages (SIMPER) analysis between the large intestines/stomachs, large intestines/jejuna, omasum-abomasums/rumens and omasum-abomasums/jejuna archaeal communities in the *Capra hircus* GITs. Avg: average dissimilarity; Contrib. (%): percent contributed; Cumul. (%): cumulative percentage; Ru: rumen; OA: omasum + abomasum; Je: jejunum; Ce: cecum; Co: colon. Bold indicates the relative percentage of the archaeal genera that contributed most to the observed dissimilarity between samples.
(PDF)
