# Supplementary figures and images for: The gastrointestinal microbiome of browsing goats (Capra hircus)
Source: PLoS One. 2022 Oct 17;17(10):e0276262. doi: 10.1371/journal.pone.0276262 (PMC9576075; doi:10.1371/journal.pone.0276262)

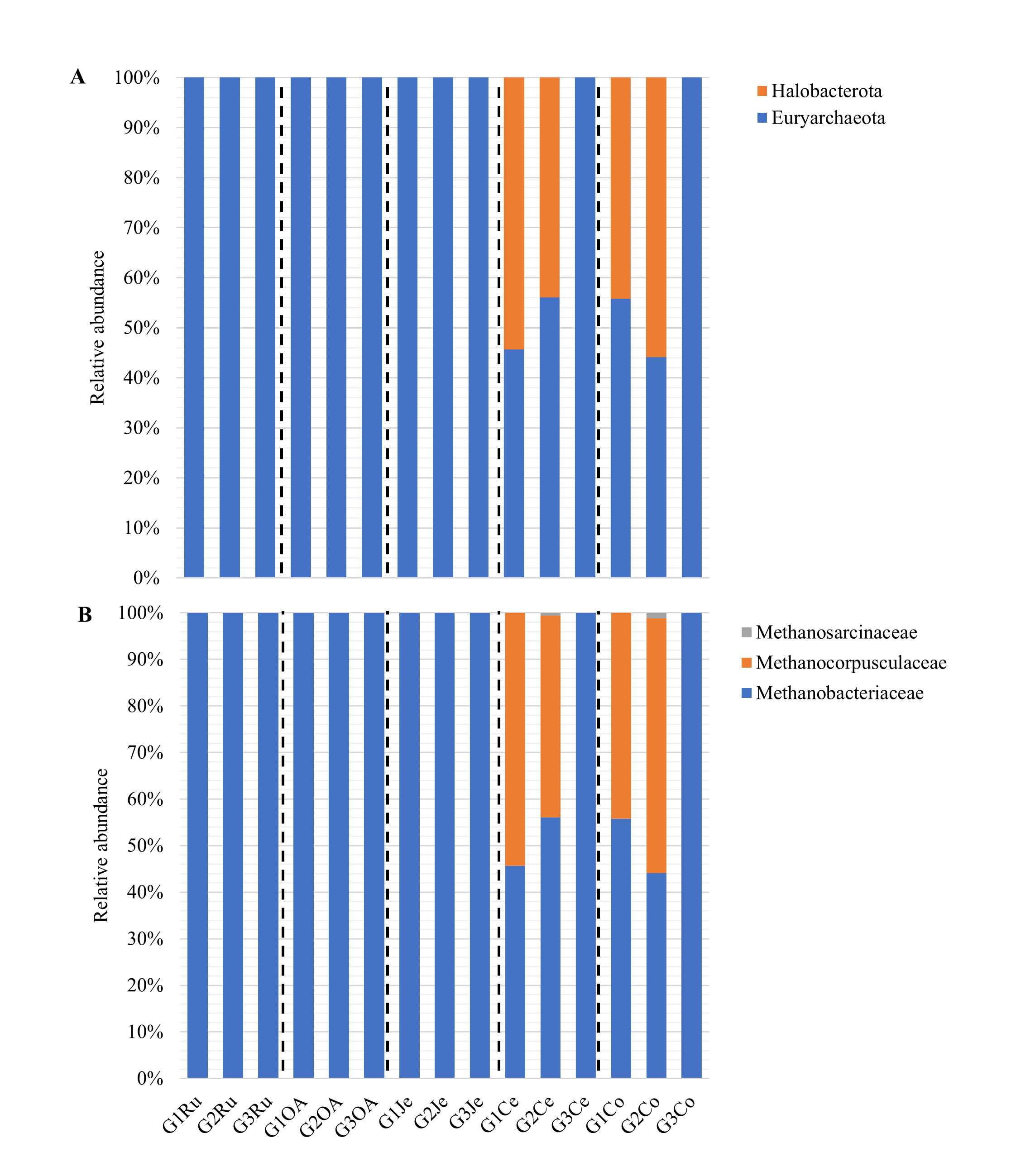

Supplement: S1 Fig — Distribution of the total archaeal phyla (A) and archaeal families (B) in Capra hircus GITs. Ru: rumen; OA: omasum + abomasum; Je: jejunum; Ce: cecum; Co: colon; G: goat. (TIF) [file pone.0276262.s001.tif]

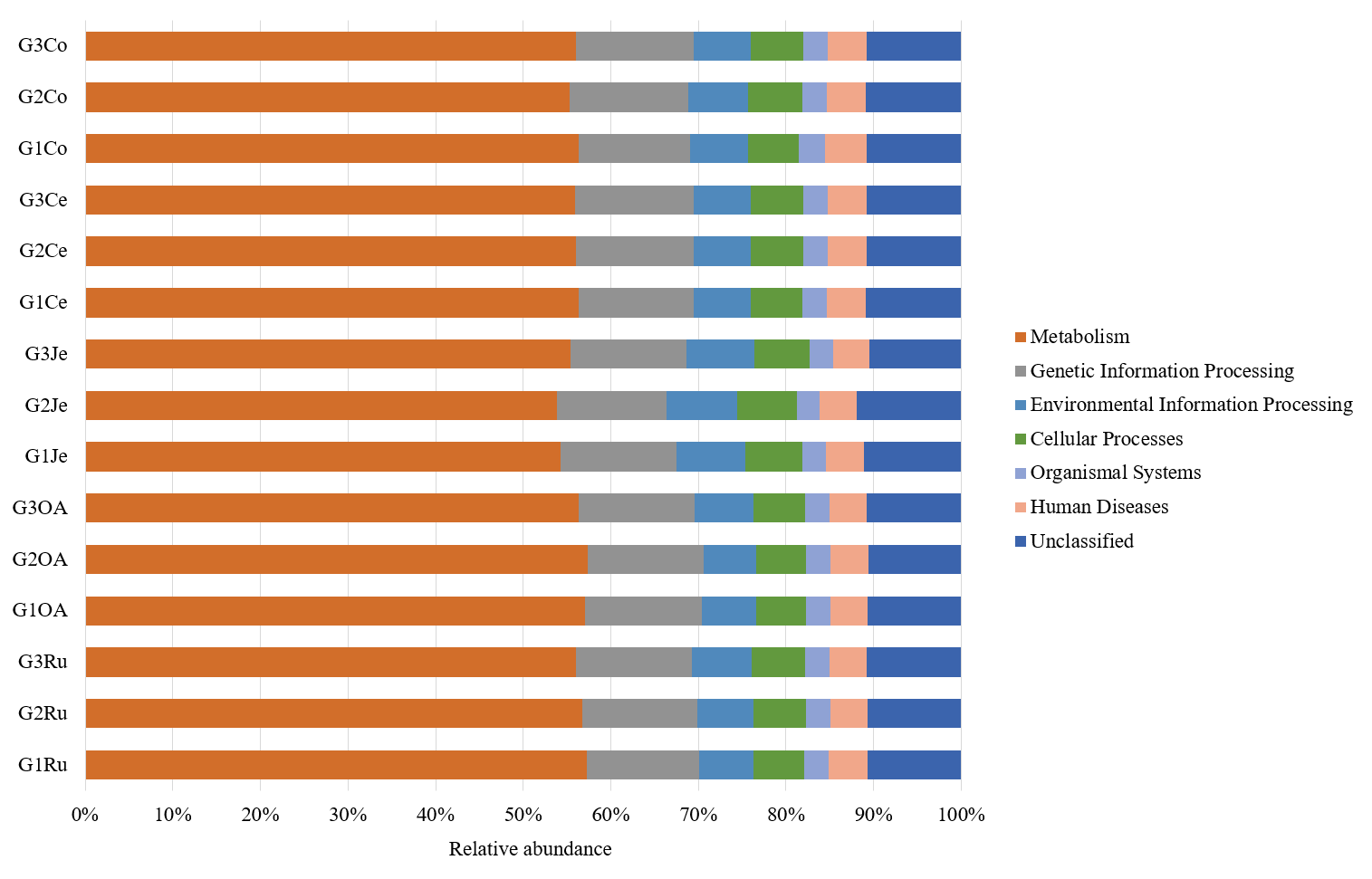

Supplement: S2 Fig — Ru: rumen; OA: omasum + abomasum; Je: jejunum; Ce: cecum; Co: colon; G: goat. (TIF) [file pone.0276262.s002.tif]

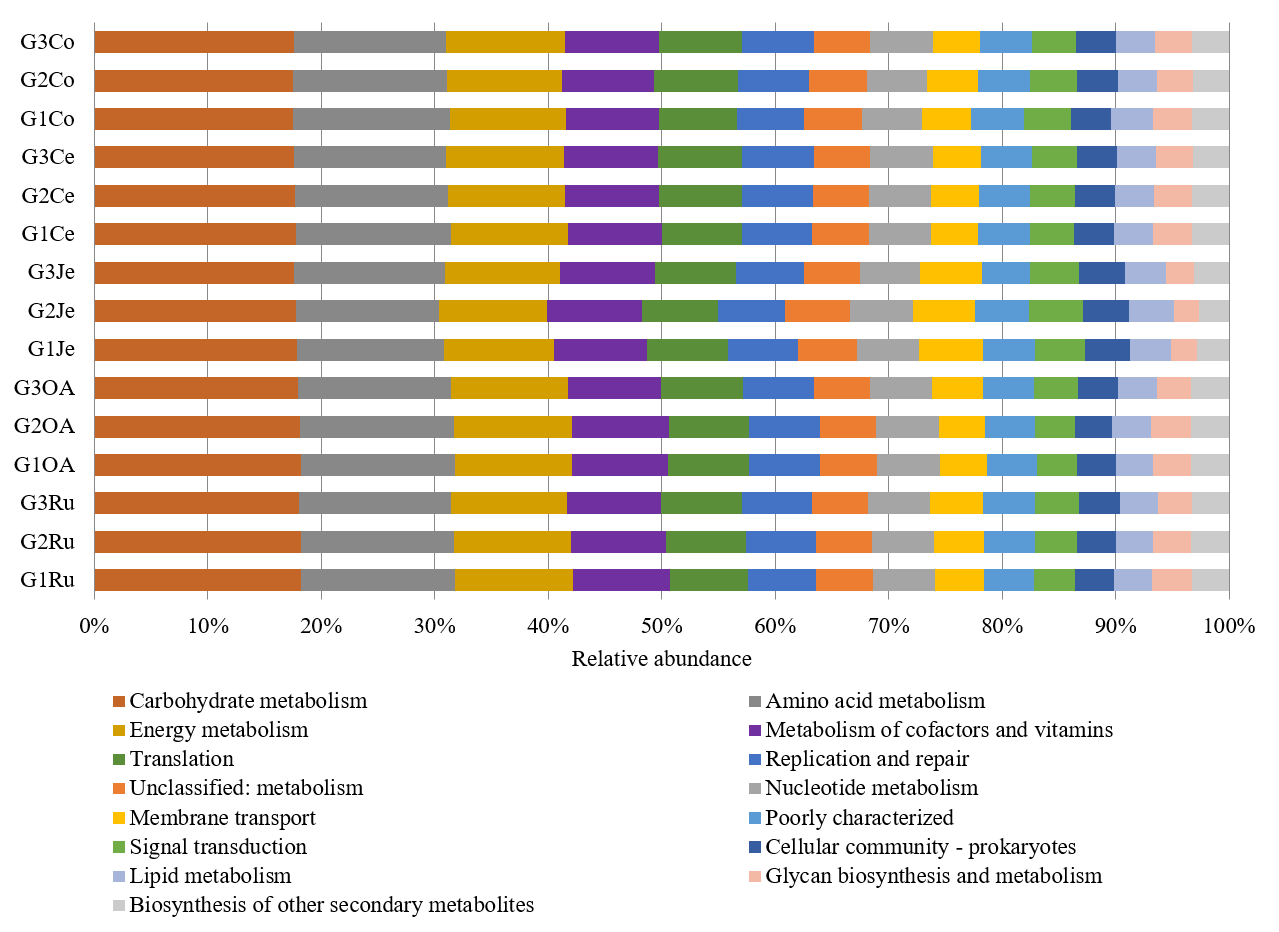

Supplement: S3 Fig — Ru: rumen; OA: omasum + abomasum; Je: jejunum; Ce: cecum; Co: colon; G: goat. (TIF) [file pone.0276262.s003.tif]

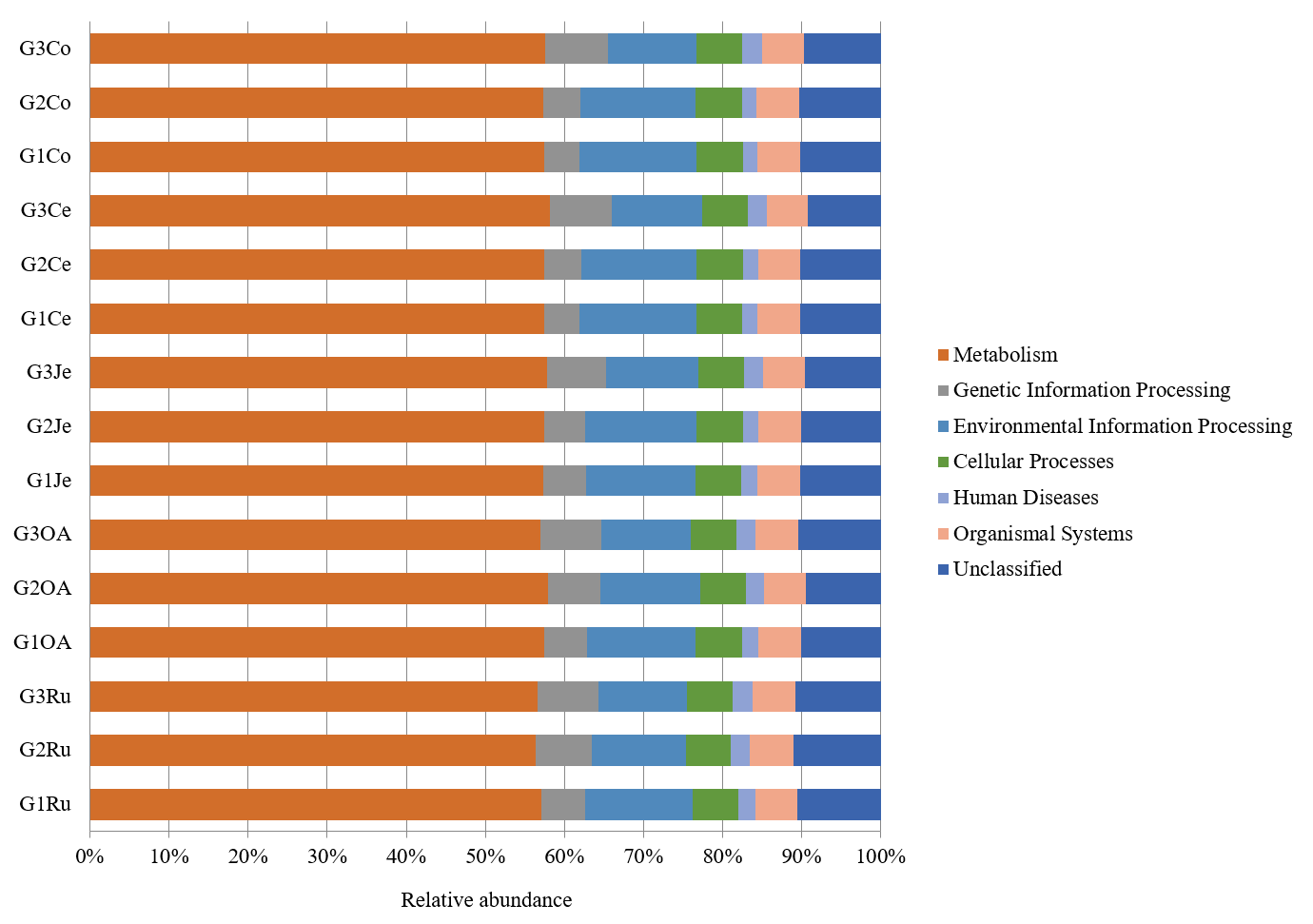

Supplement: S4 Fig — Ru: rumen; OA: omasum + abomasum; Je: jejunum; Ce: cecum; Co: colon; G: goat. (TIF) [file pone.0276262.s004.tif]

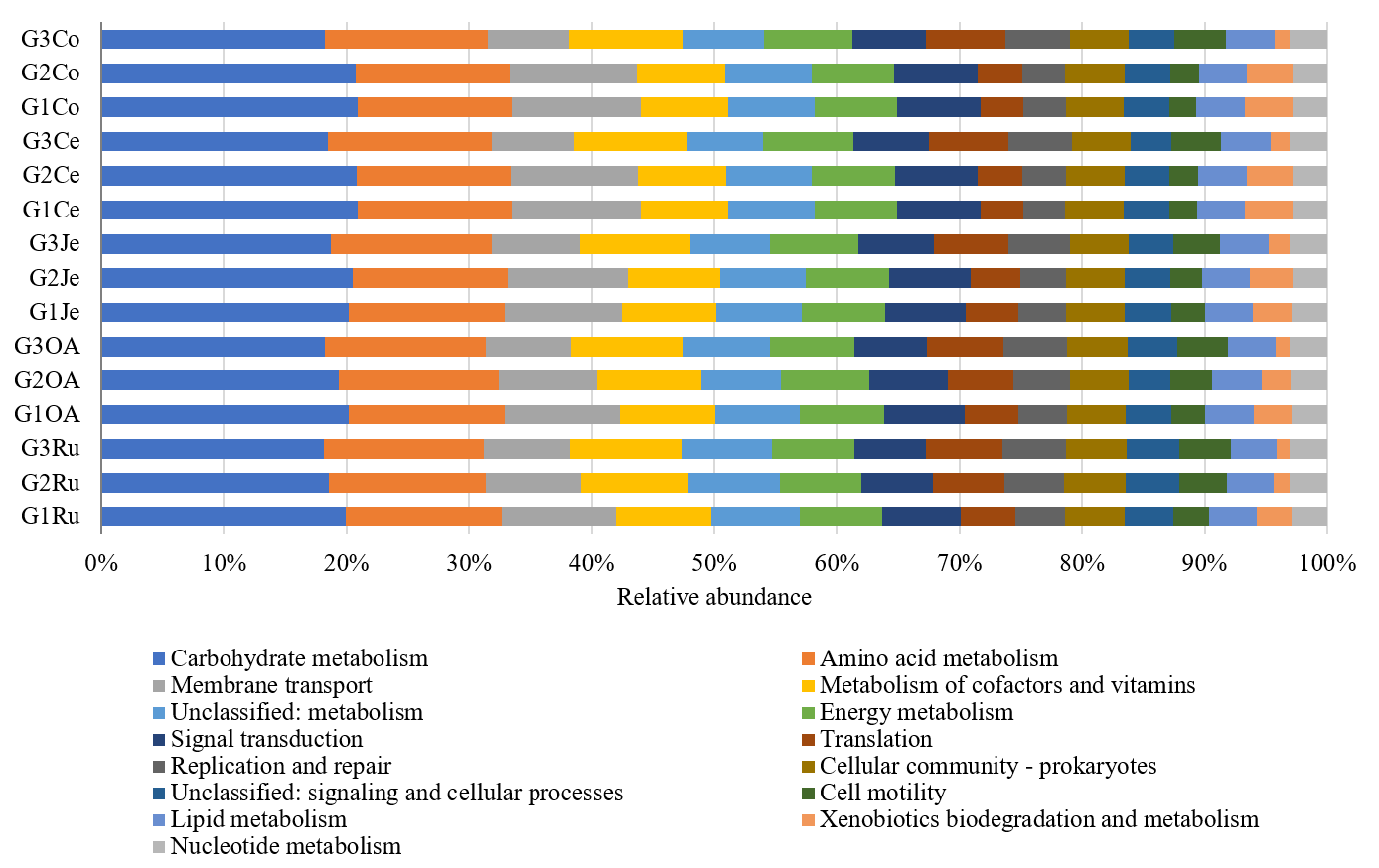

Supplement: S5 Fig — Ru: rumen; OA: omasum + abomasum; Je: jejunum; Ce: cecum; Co: colon; G: goat. (TIF) [file pone.0276262.s005.tif]

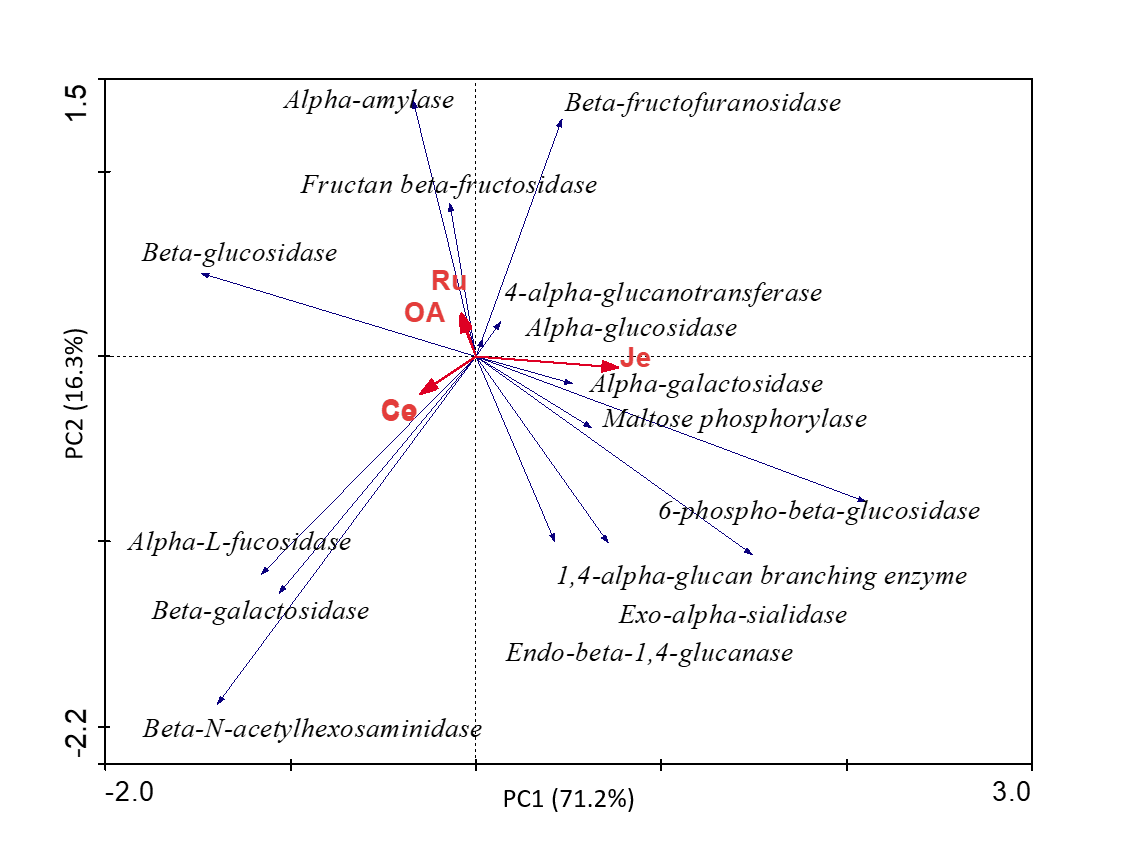

Supplement: S6 Fig — Ru: rumen; OA: omasum + abomasum; Je: jejunum; Ce: cecum; Co: colon; G: goat. (TIF) [file pone.0276262.s006.tif]
