# Supplementary material for: The gastrointestinal microbiome of browsing goats (Capra hircus)
Source: PLoS One. 2022 Oct 17;17(10):e0276262. doi: 10.1371/journal.pone.0276262 (PMC9576075; doi:10.1371/journal.pone.0276262)
Supplement: S1 Table — (PDF) [file pone.0276262.s007.pdf]

S1 Table Overall average dissimilarity (in percentage) between the bacterial communities present in the *Capra hircus* GITs.

%	Ru	OA	Je	Ce	Co
**Ru**					
**OA**	30.3				
**Je**	**78.3**	**78.8**			
**Ce**	68.2	68.0	**86.0**		
**Co**	65.4	65.4	**85.4**	28.3	

Ru: rumen; OA: omasum + abomasum; Je: jejunum; Ce: cecum; Co: colon. Bold indicates the highest average dissimilarity between the GIT sections.
