# Supplementary material for: The gastrointestinal microbiome of browsing goats (Capra hircus)
Source: PLoS One. 2022 Oct 17;17(10):e0276262. doi: 10.1371/journal.pone.0276262 (PMC9576075; doi:10.1371/journal.pone.0276262)
Supplement: S2 Table — Avg: average dissimilarity; Contrib. (%): percent contributed; Cumul. (%): cumulative percentage; Ru: rumen; OA: omasum + abomasum; Je: jejunum; Ce: cecum; Co: colon. Bold indicates the relative percentage of the bacterial genera that contributed most to the observed dissimilarity between samples. (PDF) [file pone.0276262.s008.pdf]

S2Table Similarity percentages (SIMPER) analysis between the stomachs/jejuna and large intestine/jejuna the bacterial communities in the *Capra hircus* GITs.

Ru/Je					
**Taxon**	**Avg**	**Contrib. (%)**	**Cumul. (%)**	**Mean Ru**	**Mean Je**
Romboutsia	12.3	**15.7**	15.7	0.0	24.6
Prevotella	11.3	**14.4**	30.2	23.0	0.4
WCHB1-41_ge	5.7	7.3	37.4	1.7	13.1
Rikenellaceae_RC9_gut_group	3.9	5.0	42.4	7.9	0.0
**OA/Je**					
**Taxon**	**Avg**	**Contrib. (%)**	**Cumul. (%)**	**Mean OA**	**Mean Je**
Romboutsia	12.3	**15.6**	15.6	0.0	24.6
Prevotella	10.5	**13.3**	29.0	21.4	0.4
WCHB1-41_ge	5.7	7.3	36.2	1.7	13.1
Rikenellaceae_RC9_gut_group	3.9	5.0	41.2	7.9	0.0
**Ce/Je**					
**Taxon**	**Avg**	**Contrib. (%)**	**Cumul. (%)**	**Mean Ce**	**Mean Je**
Romboutsia	12.1	**14.1**	14.1	0.4	24.6
WCHB1-41_ge	6.3	**7.3**	21.4	0.6	13.1
Rikenellaceae_RC9_gut_group	4.0	4.7	26.1	8.1	0.0
Bacteroides	4.0	4.7	30.7	8.1	0.0
**Co/Je**					
**Taxon**	**Avg**	**Contrib. (%)**	**Cumul. (%)**	**Mean Co**	**Mean Je**
Romboutsia	12.3	**14.4**	14.4	0.1	24.6
WCHB1-41_ge	6.4	**7.5**	21.8	0.3	13.1
Rikenellaceae_RC9_gut_group	4.3	5.0	26.9	8.6	0.0
Bacteroides	3.6	4.3	31.1	7.3	0.0
**Ru/Ce**					
**Taxon**	**Avg**	**Contrib. (%)**	**Cumul. (%)**	**Mean Ru**	**Mean Ce**
Prevotella	11.0	**16.2**	16.2	23.0	1.0
Bacteroides	4.0	**5.9**	22.1	0.0	8.1

UCG-010_ge	3.1	4.5	26.6	1.5	7.7
UCG-005	2.7	4.0	30.6	0.6	6.1
			**Ru/Co**		
**Taxon**	**Avg**	**Contrib. (%)**	**Cumul. (%)**	**Mean Ru**	**Mean Co**
Prevotella	11.0	**16.8**	16.8	23.0	1.0
Bacteroides	3.7	**5.6**	22.4	0.0	7.3
Alistipes	3.0	4.5	26.9	0.0	5.9
UCG-005	2.2	3.4	30.3	0.6	5.1
			**OA/Ce**		
**Taxon**	**Avg**	**Contrib. (%)**	**Cumul. (%)**	**Mean AO**	**Mean Ce**
Prevotella	10.2	**15.0**	15.0	21.4	1.0
Bacteroides	4.0	**5.9**	20.9	0.0	8.1
UCG-010_ge	3.1	4.6	25.5	1.4	7.7
Alistipes	2.5	3.7	29.3	0.0	5.1
			**OA/Co**		
**Taxon**	**Avg**	**Contrib. (%)**	**Cumul. (%)**	**Mean AO**	**Mean Co**
Prevotella	10.2	**15.6**	15.6	21.4	1.0
Bacteroides	3.7	**5.6**	21.2	0.0	7.3
Alistipes	3.0	4.5	25.7	0.0	5.9
Muribaculaceae_ge	2.5	3.8	29.5	5.1	0.1

Avg: average dissimilarity; Contrib. (%): percent contributed; Cumul. (%): cumulative percentage; Ru: rumen; OA: omasum + abomasum; Je: jejunum; Ce: cecum; Co: colon. Bold indicates the relative percentage of the bacterial genera that contributed most to the observed dissimilarity between samples.
