# Supplementary material for: The gastrointestinal microbiome of browsing goats (Capra hircus)
Source: PLoS One. 2022 Oct 17;17(10):e0276262. doi: 10.1371/journal.pone.0276262 (PMC9576075; doi:10.1371/journal.pone.0276262)
Supplement: S3 Table — Not Classified (N.C.). *Enzymes reported among the main to be involved in the direct degradation of lignocellulose [73, 92]. (PDF) [file pone.0276262.s009.pdf]

Table S3. Sequence based classification of Glycoside Hydrolases (GHs) predicted through 16S gene sequences by PICRUSt2 in the bacterial communities of *Capra hircus* GITs.

EC number	Enzyme	Families
EC:2.4.1.10	Levansucrase	32,68
EC:2.4.1.161	Oligosaccharide 4-alpha-D-glucosyltransferase	31
EC:2.4.1.18	1,4-alpha-glucan branching enzyme	13,57
EC:2.4.1.20*	Cellobiose phosphorylase	94
EC:2.4.1.211	1,3-beta-galactosyl-N-acetylhexosamine phosphorylase	112
EC:2.4.1.230	Kojibiose phosphorylase	65
EC:2.4.1.247	Beta-D-galactosyl-(1->4)-L-rhamnose phosphorylase	112
EC:2.4.1.25	4-alpha-glucanotransferase	13,57,77
EC:2.4.1.279	Nigerose phosphorylase	65
EC:2.4.1.280	N,N'-diacetylchitobiose phosphorylase	94
EC:2.4.1.281	4-O-beta-D-mannosyl-D-glucose phosphorylase	130
EC:2.4.1.319	Beta-1,4-mannooligosaccharide phosphorylase	130
EC:2.4.1.320	1,4-beta-mannosyl-N-acetylglucosamine phosphorylase	130
EC:2.4.1.321	Cellobionic acid phosphorylase	94
EC:2.4.1.4	Amylosucrase	13
EC:2.4.1.5	Dextransucrase	70
EC:2.4.1.64	Alpha,alpha-trehalose phosphorylase	65
EC:2.4.1.7	Sucrose phosphorylase	13
EC:2.4.1.8	Maltose phosphorylase	65
EC:3.1.1.73*	Feruloyl esterase	10,62,78
EC:3.2.1.1	Alpha-amylase	13,57,126
EC:3.2.1.10	Oligo-1,6-glucosidase	13,31
EC:3.2.1.11	Dextranase	31,49,66
EC:3.2.1.113	Mannosyl-oligosaccharide 1,2-alpha-mannosidase	38,47,92
EC:3.2.1.122	Maltose-6'-phosphate glucosidase	4
EC:3.2.1.123	Endoglycosylceramidase	5
EC:3.2.1.130	Glycoprotein endo-alpha-1,2-mannosidase	99
EC:3.2.1.132	Chitosanase	3,5,8,46,75,80
EC:3.2.1.135	Neopullulanase	13
EC:3.2.1.136	Glucuronoarabinoxylan endo-1,4-beta-xylanase	30
EC:3.2.1.139*	Alpha-glucuronidase	67
EC:3.2.1.14	Chitinase	18,19,23
EC:3.2.1.141	4-alpha-D-((1->4)-alpha-D-glucano)trehalose trehalohydrolase	13
EC:3.2.1.156	Oligosaccharide reducing-end xylanase	8,1
EC:3.2.1.165	Exo-1,4-beta-D-glucosaminidase	2,9,35
EC:3.2.1.17	Lysozyme	18,19,22,23,24, 25,73,108,NC
EC:3.2.1.170	Mannosylglycerate hydrolase	63
EC:3.2.1.172	Unsaturated rhamnogalacturonyl hydrolase	105
EC:3.2.1.177	Alpha-D-xyloside xylohydrolase	31
EC:3.2.1.18	Exo-alpha-sialidase	33,34,83,156,NC

EC:3.2.1.180	Unsaturated chondroitin disaccharide hydrolase	88
EC:3.2.1.185	Non-reducing end beta-L-arabinofuranosidase	127,142,143,146
EC:3.2.1.187	(Ara-f)(3)-Hyp beta-L-arabinobiosidase	121
EC:3.2.1.20*	Alpha-glucosidase	4,13,31,76,97,122
EC:3.2.1.21*	Beta-glucosidase	1,2,3,5,16,30,39,116, 131,NC
EC:3.2.1.22*	Alpha-galactosidase	4,27,31,36,57,97,110
EC:3.2.1.23	Beta-galactosidase	1,2,3,5,16,35,42,50,54, 147,165,173,NC
EC:3.2.1.24	Alpha-mannosidase	38,92
EC:3.2.1.25*	Beta-mannosidase	1,2,5,113,164
EC:3.2.1.26	Beta-fructofuranosidase	32,68,100
EC:3.2.1.28	Alpha,alpha-trehalase	13,15,37,65
EC:3.2.1.3	Glucan 1,4-alpha-glucosidase	15,97
EC:3.2.1.31	Beta-glucuronidase	1,2,3,79,137
EC:3.2.1.35	Hyaluronoglucosaminidase	16,56,84
EC:3.2.1.37*	Xylan 1,4-beta-xylosidase	1,2,3,5,10,30,39,43,52, 54,120
EC:3.2.1.4*	Cellulase (or endo-β-1,4-glucanase)	5,6,7,8,9,10,12,26,44, 45,48,51,74,124,148,NC
EC:3.2.1.40	Alpha-L-rhamnosidase	33,78,106
EC:3.2.1.41	Pullulanase	13,57
EC:3.2.1.45	Glucosylceramidase	3,5,116
EC:3.2.1.50	Alpha-N-acetylglucosaminidase	89
EC:3.2.1.51	Alpha-L-fucosidase	29,95,151,NC
EC:3.2.1.52	Beta-N-acetylhexosaminidase	2,3,18,20,84
EC:3.2.1.55*	Non-reducing end alpha-L-arabinofuranosidase	2,3,10,39,43,51,54,62
EC:3.2.1.58	Glucan 1,3-beta-glucosidase	5,16,55
EC:3.2.1.64	2,6-beta-fructan 6-levanbiohydrolase	32
EC:3.2.1.65	Levanase	32
EC:3.2.1.68	Isoamylase	13
EC:3.2.1.70	Glucan 1,6-alpha-glucosidase	13,15
EC:3.2.1.73	Licheninase	5,8,9,11,12,16,17, 26,NC
EC:3.2.1.78*	Mannan endo-1,4-beta-mannosidase	5,26,44,45,113,134
EC:3.2.1.8*	Endo-1,4-beta-xylanase	3,5,6,8,10,11,26,30, 43,98
EC:3.2.1.80	Fructan beta-fructosidase	32
EC:3.2.1.81	Beta-agarase	16,50,86,118
EC:3.2.1.82	Exo-poly-alpha-galacturonosidase	28
EC:3.2.1.85	6-phospho-beta-galactosidase	1
EC:3.2.1.86	6-phospho-beta-glucosidase	1,4
EC:3.2.1.89*	Arabinogalactan endo-beta-1,4-galactanase	53,147
EC:3.2.1.91*	Cellulose 1,4-beta-cellobiosidase (non-reducing end) (or cellobiohydrolase)	5,6,9,51
EC:3.2.1.93	Alpha,alpha-phosphotrehalase	13
EC:3.2.1.96	Mannosyl-glycoprotein endo-beta-N-acetylglucosaminidase	18,20,73,85
EC:3.2.1.97	Endo-alpha-N-acetylgalactosaminidase	101
EC:3.2.1.99*	Arabinan endo-1,5-alpha-L-arabinosidase	43

EC:3.5.2.17	Hydroxyisourate hydrolase	1
EC:4.2.2.17	Inulin fructotransferase (DFA-I-forming)	91
EC:5.4.99.15	(1->4)-alpha-D-glucan 1-alpha-D-glucosylmutase	13
EC:5.4.99.16	Maltose alpha-D-glucosyltransferase	13

Not Classified (N.C.). *Enzymes reported among the main to be involved in the direct degradation of lignocellulose [73,92]

References

73. Saini A, Aggarwal NK, Sharma A, Yadav A. Actinomycetes: A source of lignocellulolytic enzymes. Enzyme Res. 2015;2015:1–15. doi: 10.1155/2015/279381

92. Sweeney MD, Xu F. Biomass converting enzymes as industrial biocatalysts for fuels and chemicals: recent developments. Catalysts . 2012;2(2):244–63. doi: 10.3390/catal2020244
