# Supplementary material for: The gastrointestinal microbiome of browsing goats (Capra hircus)
Source: PLoS One. 2022 Oct 17;17(10):e0276262. doi: 10.1371/journal.pone.0276262 (PMC9576075; doi:10.1371/journal.pone.0276262)
Supplement: S4 Table — Glycoside Hydrolase (GH), Glycosyl transferase (GT), and Carbohydrate Binding Module (CBM). (PDF) [file pone.0276262.s010.pdf]

S4 Table Sequence based classification of carbohydrate active enzymes (CAE) predicted through 16S gene sequences by PICRUSt2 in the archaeal communities of *Capra hircus* GITs.

**EC number**	**Enzyme**	**Classification**	
EC:2.4.1.1	Glycogen phosphorylase	GT	
EC:2.4.1.15	Alpha,alpha-trehalose-phosphate synthase (UDP-forming)	GT	
EC:2.4.1.227	acetylglucosaminyltransferase	GT	
EC:2.4.1.83	Dolichyl-phosphate beta-D-mannosyltransferase	GT	
EC:2.4.99.18	Dolichyl-diphosphooligosaccharide--protein glycotransferase	GT	
EC:3.1.3.12	Trehalose-phosphatase	GT	
EC:3.2.1.1	Alpha-amylase	GH	CBM
EC:3.2.1.4	Cellulase	GH	CBM

Glycoside Hydrolase (GH), Glycosyl transferase (GT), and Carbohydrate Binding Module (CBM).
