# Supplementary material for: The gastrointestinal microbiome of browsing goats (Capra hircus)
Source: PLoS One. 2022 Oct 17;17(10):e0276262. doi: 10.1371/journal.pone.0276262 (PMC9576075; doi:10.1371/journal.pone.0276262)
Supplement: S6 Table — Avg: average dissimilarity; Contrib. (%): percent contributed; Cumul. (%): cumulative percentage; Ru: rumen; OA: omasum + abomasum; Je: jejunum; Ce: cecum; Co: colon. Bold indicates the relative percentage of the archaeal genera that contributed most to the observed dissimilarity between samples. (PDF) [file pone.0276262.s012.pdf]

S6 Table Similarity percentages (SIMPER) analysis between the large intestines/stomachs, large intestines/jejuna, omasum-abomasums/rumens (50.3%) and omasum-abomasums/jejuna archaeal communities in the *Capra hircus* GITs.

Ru/Ce

Taxon	Avg	Contrib. (%)	Cumul. (%)	Mean Ru	Mean Ce
Methanobrevibacter	16.4	**50.0**	50.0	99.8	66.9
Methanocorpusculum	16.3	**49.5**	99.4	0.0	32.6
Methanimicrococcus	0.1	0.3	99.7	0.0	0.2
Methanosphaera	0.1	0.3	100.0	0.2	0.3
Methanocorpusculaceae _unclassified	0.0	0.0	100.0	0.0	0.0

Ru/Co

Taxon	Avg	Contrib. (%)	Cumul. (%)	Mean Ru	Mean Co
Methanobrevibacter	16.7	**50.0**	50.0	99.8	66.3
Methanocorpusculum	16.5	**49.2**	99.1	0.0	32.9
Methanimicrococcus	0.2	0.6	99.7	0.0	0.4
Methanosphaera	0.1	0.2	99.9	0.2	0.3
Methanocorpusculaceae _unclassified	0.0	0.0	100.0	0.0	0.0

OA/Ce

Taxon	Avg	Contrib. (%)	Cumul. (%)	Mean OA	Mean Ce
Methanocorpusculum	16.3	**49.3**	49.3	0.0	32.6
Methanobrevibacter	16.2	**49.0**	98.3	98.8	66.9
Methanosphaera	0.5	1.4	99.7	1.2	0.3
Methanimicrococcus	0.1	0.3	100.0	0.0	0.2
Methanocorpusculaceae _unclassified	0.0	0.0	100.0	0.0	0.0

OA/Co

Taxon	Avg	Contrib. (%)	Cumul. (%)	Mean OA	Mean Co
Methanobrevibacter	16.5	**49.0**	49.0	98.8	66.3
Methanocorpusculum	16.5	**49.0**	98.0	0.0	32.9
Methanosphaera	0.5	1.4	99.3	1.2	0.3
Methanimicrococcus	0.2	0.6	99.9	0.0	0.4
Methanocorpusculaceae _unclassified	0.0	0.0	100.0	0.0	0.0

Je/Ce					
**Taxon**	**Avg**	**Contrib. (%)**	**Cumul. (%)**	**Mean Je**	**Mean Ce**
Methanobrevibacter	16.3	**49.5**	49.5	99.3	66.9
Methanocorpusculum	16.3	**49.4**	98.9	0.0	32.6
Methanosphaera	0.2	0.7	99.7	0.7	0.3
Methanimicrococcus	0.1	0.3	100.0	0.0	0.2
Methanocorpusculaceae _unclassified	0.0	0.0	100.0	0.0	0.0

Je/Co					
**Taxon**	**Avg**	**Contrib. (%)**	**Cumul. (%)**	**Mean Je**	**Mean Co**
Methanobrevibacter	16.6	**49.5**	49.5	99.3	66.3
Methanocorpusculum	16.5	**49.1**	98.6	0.0	32.9
Methanosphaera	0.2	0.7	99.3	0.7	0.3
Methanimicrococcus	0.2	0.6	99.9	0.0	0.4
Methanocorpusculaceae _unclassified	0.0	0.0	100.0	0.0	0.0
Methanobacteriaceae _unclassified	0.0	0.0	100.0	0.0	0.0

Avg: average dissimilarity; Contrib. (%): percent contributed; Cumul. (%): cumulative percentage; Ru: rumen; OA: omasum + abomasum; Je: jejunum; Ce: cecum; Co: colon. Bold indicates the relative percentage of the archaeal genera that contributed most to the observed dissimilarity between samples.
